# An enhanced genetic algorithm solution for itinerary recommendation considering various constraints

**DOI:** 10.7717/peerj-cs.2340

**Published:** 2024-10-02

**Authors:** Muhammed Şehab, Metin Turan

**Affiliations:** 1 Computer Technology Department, Avrupa Vocational School, Kocaeli Health and Technology University, Kocaeli, Turkey; 2 Computer Engineering Department, Istanbul Ticaret University, Istanbul, Turkey

**Keywords:** Fitness function, Crossover method, Mutation ratio, Genetic algorithm, Itinerary recommendation, Optimal itinerary

## Abstract

This paper addresses the challenging task of itinerary recommendation for tourists and proposes an approach for suggesting efficient optimal itineraries in Istanbul, based on constraints. The paper presents an enhanced version of the genetic algorithm (GA), which aims to optimize the itineraries considering various constraints and preferences of the tourists. The improvement of the GA involved suggesting a customized fitness function tailored to address the complexities of the tourism problem, considering factors such as distance, time, cost, tourists’ budget, and their desired activities and attractions. Additionally, we proposed a new crossover method, named “Copy Order Crossover” and we modified the tournament selection method beside enhancing the implementation of the swap mutation method for greater efficiency and adaptability. The enhanced GA is evaluated on the Burma dataset taken from TSPLIB, and our constructed Istanbul dataset, achieving significant enhancement rates in GA (43.89% for Istanbul, and 56.60% for Burma). This paper provides a detailed account of the proposed approach, its implementation, and the evaluation conducted. The experimental results conclusively demonstrated the superiority of the proposed approach over alternative methods in terms of time, efficiency, and accuracy. This paper finishes with an outlook with a detailed potential approach to overcome itinerary recommendation problem limitations.

## Introduction

Tourism is a major industry that contributes significantly to the economy of many countries. One of the critical aspects of tourism is itinerary recommendation. Tourists want to visit as many places as possible within a limited time, while also minimizing travel time and expenses. Traditionally, itinerary recommendation is done manually, which can be time-consuming and may not always lead to optimal itineraries. However, with the recent advancements in machine learning and optimization approaches, it is now possible to automate itinerary recommendation. Like using deep learning in the itinerary recommendation system proposed by Chen et al. (2021) to identify landmarks from pictures taken by tourists, allowing them to retrieve information and plan itineraries. Sentiment analysis and geo-tagging were used effectively by Bapat et al. (2022) to recommend many activities and destinations in the real-time. One of the most used approaches in this domain is the GA. Genetic algorithms (GAs) are a computational optimization technique inspired by the principles of natural selection and genetics. As a subset of evolutionary algorithms (EAs), GAs employ biologically inspired processes such as initialization, selection, crossover, and mutation to develop high-quality solutions for complex optimization and search problems (Albadr et al., 2020). GAs have a wide range of applications across various fields. Here are some key areas where they may be used:

In the field of biology, GAs are widely used for optimization in evolutionary biology, including phylogeny construction and modeling biological systems (Alhijawi & Awajan, 2024). In the analysis of cancer datasets and biological data, they assist in parameter estimation for fitness functions for improving the accuracy of biological models (Xu & Jackson, 2019). GAs are increasingly being utilized to optimize genetic and neural network models for various biological applications (Pandi et al., 2022).

In medicine, GAs offer optimal solutions for many medical problems including disease screening, diagnosis, treatment planning, and overall healthcare management (Ghaheri et al., 2015; Sharma & Kumar, 2022). They were used in medical diagnosis like diabetes by improving the accuracy of the diagnostic model throughout optimizing neural network weights (Karmakar, 2022). GAs also contribute to the field of pharmacovigilance and prognosis, aiding in the development of personalized medicine and tailored treatment plans (Myszczynska et al., 2020).

In remote sensing, GAs are used for band selection in hyperspectral remote sensing to mitigate the curse of dimensionality, optimizing the selection of spectral bands for better data analysis (Ma et al., 2003; Saqui et al., 2016). GAs enhance classification accuracy in hyperspectral image processing by optimizing parameters for classifiers like support vector machines (SVM) (Lin & Zhang, 2020; Wang & Luo, 2023). They are also applied in image registration and resource scheduling for Earth Observation Satellites (EOS), improving the efficiency of remote sensing operations (Wu et al., 2021; Xing et al., 2024).

In finance networks, GAs offer solutions for tasks ranging from market forecasting and portfolio optimization to credit risk assessment in supply chain finance (Sang, 2021). GAs are employed in optimizing financial databases and training neural networks for making decision (Li & Shi, 2022). Notably, the integration of GAs with artificial neural networks has demonstrated significant promise in enhancing the accuracy of financial predictions and risk assessments (Sharma et al., 2022; Li, Li & Wu, 2024).

In logistics, GAs optimize logistics paths, facility locations, and resource allocation in both commercial and humanitarian logistics (He, Tang & Abdullah, 2022). They improve distribution methods, ensuring efficient delivery and resource management (Xin, Xu & Manyi, 2022; Maroof, Ayvaz & Naeem, 2024), and GAs are applied in emergency logistics to generate feasible schedules for disaster relief (Chang et al., 2014; Razavi et al., 2021). GAs were utilized effectively in Green Capacitated Arc Routing Problem (G-CARP) by Tirkolaee et al. (2018) to minimize the total cost of transportation, including greenhouse gas emissions, vehicle usage, and routing. For calculating greenhouse gas emissions, their model considered factors such as vehicle speed, weather conditions, and load on the vehicle. Itinerary recommendation using GA is a promising approach for the tourism industry. The GA generates a population of candidate solutions, where each solution represents a possible itinerary. The choice of superior metrics in a GA depends on the satisfaction of the tourist’s constraints. In another words, in instances where a tourist’s considerations encompass travel distance, time, and cost concurrently, these variables have to be amalgamated within a singular metric, such as the fitness metric delineated in this research.

Conversely, when the tourist’s concern pertains solely to distance, the fitness metric becomes contingent solely upon this parameter. Table 1 in the Methodology section introduces the superior metrics used in this research for itinerary recommendation. The GA then evolves the population of candidate solutions through selection, crossover, and mutation operations until a satisfactory solution is found (Goldberg, 1989; Katoch, Chauhan & Kumar, 2021).

**Table 1:** Superior metrics for optimal itinerary.

**Metric**	**Description**
Fitness function	Evaluates the quality of an itinerary based on factors like travel time, cost, and travel distance.
Travel distance	Quantifies the travel distance between attractions and aims to minimize it in the generated itineraries.
Travel time	Quantifies the time spent traveling between attractions and aims to minimize it in the generated itineraries.
Travel cost	Quantifies the traveling cost between attractions in order to minimize it in the generated itineraries and to ensure not exceeding the tourist budget.
Individual diversity (Entropy)	In our study, we observed that higher fitness values corresponds to lower entropy, indicating a more optimal itinerary. Itineraries with superior fitness demonstrate reduced diversity, where all places from the same neighborhood are adjacent in the sequence. As it’s seen in Table 2 and later in the Experimental Results section, the “ABCDDDDDEEEF” sequence with 5 different places from the D neighborhood, like Hagia Sophia Neighborhood places (10–9–7–11–8), and 3 different places from the E neighborhood appearing consecutively, exhibit high fitness value and low entropy. Conversely, itineraries with higher diversity, such as “ADBDCDEDEFDE,” are less optimal, resulting in lower fitness values.
Population diversity	When the population individuals are congruent, then its diversity is low and the probability of selecting two different individuals for producing better offspring becomes nearly impossible (remains in local mostly) and the GA process will slow down and may not reach the global optimal solution. In the opposite case, when the population individuals are diverse, the GA benefits from a wider solution space and higher chances of selecting superior offspring, leading to a more efficient convergence towards the global optimal solution.

The potential benefits of using GA for itinerary recommendation include optimizing travel time and expenses while handling large-scale itinerary recommendation problems. The algorithm can generate a large number of candidate solutions and evolve them to find the best solution in a reasonable amount of time. By considering the tourist’s preferred destinations and budget, many researchers like Cao (2022) who utilized the GA algorithm to generate itineraries that minimize distances, travel time, and costs.

This paper introduces an enhanced GA for itinerary recommendation in tourism. The Related Work section provides a comprehensive overview of existing itinerary recommendation techniques, with a particular focus on GA-based approaches. It delves into the details of GA steps, including selection and crossover methods. The Methodology section then presents a mathematical formulation of the problem. We detail our proposed GA approach for itinerary recommendation, which includes setting up information matrices, creating an initial population, proposing a novel fitness function for evaluating algorithm outputs, and modifications to the tournament selection method and the introduction of a new crossover method. Finally, the mutation operator used in the GA is described. The Experimental Results section presents the findings of our experiments, including statistical analysis, performance evaluation, and a comparison with existing approaches. We conclude the paper by summarizing the key findings of this study and outlining future directions for developing some proposed model.

## Related Work

Itinerary recommendation problem is parallel to Travelling Salesman Problem (TSP) and Vehicle Route Problem (VRP) which are solved using GAs and some other methods as well. TSP, VRP, and itinerary recommendation problems are solve using many other methods other than GA. Zhang & Tang (2018) utilized robust optimization method to address uncertainties in travel time and deadlines in public transportation, maximizing the size of the uncertainty set to find the most reliable route ensuring arrival by the deadline even under unpredictable travel times within a certain range. Ding et al. (2024) proposed a two-stage stochastic model for reliable tourist itineraries. It considers traffic uncertainties, tourist preferences, and time limits, resulting in high reliability, improved tourist satisfaction, and increased tour operator profits. Chen et al. (2023) proposed an improved Ant Colony System for travel itineraries that considers attractions, restaurants, and hotels with user satisfaction. Their method allows revisiting locations and avoids getting stuck in repetitive loops.

The utilization of GA for determining an optimal tourist route based on various constraints has become prevalent in the field. (Choi et al., 2022) in their research, effectively utilized GAs with an extra fine-tune operation for addressing the problem of multi-day itinerary generation with time windows of destinations. Kaleli, Bayğın & Naralan (2022) applied GA, the clonal selection algorithm, and DNA computing algorithms to find optimal routes for municipal bus lines in Erzurum. This resulted in recommendations for shorter paths. Paulavičius et al. (2023) proposed a novel approach to Personalized Tourism Recommendation Systems (TRS) that addresses the complexities of itinerary creation by considering factors like time constraints, mandatory visits, and user preferences. They developed a greedy GA to efficiently find optimal or near-optimal solutions. Their findings demonstrate significant effectiveness compared to existing methods. The GA process involves several fundamental steps. Firstly, an initial population of potential solutions is randomly generated, representing different tourist destinations. The selection of suitable parent individuals from this population, followed by the application of crossover, produces new offspring solutions. The quality of selected parents plays a crucial role in influencing the fitness of subsequent generations. If one or both parents exhibit high fitness values, the resulting offspring individuals are likely to possess better fitness values, provided an effective crossover method is employed.

Numerous selection methods are available, with random selection being the simplest. In this approach, two parents are chosen randomly from the entire population. However, this method may not yield the optimal solution or may require additional iterations to increase the probability of selecting parents with higher fitness values. The tournament selection method enhanced that probability by selecting a group of *N* individuals randomly with a tournament group size *N* > 2. The two individuals with the highest fitness values within this tournament group are then designated as the winning parents for crossover (Fang & Li, 2010). Tournament selection method is used widely with the GA by many researchers like (Prayudani et al., 2020) who investigated in their study how tournament selection method affects the GA for solving TSP. Although this method is more time-consuming, it increases the likelihood of selecting individuals with superior fitness. Nonetheless, the tournament selection method may become ineffective if, in a particular generation, the tournament group exhibits low fitness values or consists of highly similar individuals. Consequently, subsequent generations may experience little change without approaching the optimal solution and remain in the local solution.

The effectiveness of the crossover method in generating high-quality solutions remains significant, even if the selection method is suboptimal. However, when the selection method is inadequate, it may necessitate additional time to obtain the best solution. This drawback can be mitigated by introducing an efficient mutation rate at appropriate intervals during the GA process, thereby preventing monotony at advanced stages. Consequently, both parents and their offspring coexist in the subsequent generation, arranged in descending order based on their fitness values.

To maintain a consistent population size across all generations, the least fit individuals are pruned. Our objective is to achieve the highest fitness value within the shortest timeframe. In other words, by refining and optimizing the GA process, we aim to obtain the optimal solution in the earliest possible generation, thereby minimizing the elapsed time required.

The most applied crossover method is the Cycle Crossover (CX) which was introduced by Oliver, Smith & Holland (1987) and was efficiently utilized in many fields, like Travelling Salesman Problem TSP by Singh et al. (2022). In this method random cycles are selected from parent sequences to be retained in their positions, the other elements outside these cycles are swapped between parents.

Partially Mapped Crossover (PMX) by Goldberg (1985) is another widely used method. Desjardins et al. (2017) applied it in their evolutionary multi-objective optimization algorithm to suggest multiple sensor relocation trajectories to the network manager. A random substring from each parent is selected to be swapped in the offspring sequences, then places duplication problem can be fixed by mapping them to their corresponding in the other offspring sequence.

The Order Crossover Method (OX) by Davis (1985) and Linear Order Crossover (LOX) by Gen & Cheng (1999) were applied in lot of studies like Vehicle Routing Problem (VRP) by Hvattum (2022) and TSP by Toathom & Champrasert (2022). Both of those methods swap the middle randomly selected parts and then they fix the duplication problem by considering the two subsequence edges as one linear sequence either by starting to remove the duplicated places from the second cut point like in OX or starting from the very beginning in each sequence like in LOX.

Our proposed Copy Order Crossover (COX) method doesn’t swap the randomly selected middle subsequence parts so there will not be any duplication problem in the offsprings. Additionally, it does not consider the two subsequence edges as one linear sequence like the other order methods. Each of the subsequence edges places will be reordered apparently in the offsprings as their places order in the other parent. This proposed crossover method guarantees producing offspring sequences which inherit their features from both of their parents without completely destroying the order of the places, unlike other methods for fixing duplication problems and reordering the places. The Crossover Step section explains COX in detail. To show the efficiency of this method we applied GA with CX and with COX and then we made many comparisons as it appears in the Experimental Results section. The mentioned crossover methods are explained in the Crossover Step section with some numerical example taken from our case study dataset.

## Methodology

To understand tourist priorities, we interviewed a sample of tourism experts in Istanbul with direct experience in customer service. These experts indicated that budget, trip duration, and travel distance were primary concerns for most tourists. In line with these findings, our study aims to optimize itineraries by minimizing these factors, as detailed in the problem’s mathematical formulation.

### The mathematical formulation

The itinerary recommendation problem for a tourist planning to visit *n* different places can be modeled as an itinerary graph *R* = (*P*, *E*), where:

• $P= \left\{ 1,2,\ldots ,n \right\} $ is the set of tourist destination places.
• *E*⊆(*i*, *j*):*i*, *j* ∈ *P*, *i* ≠ *j* is the set of edges representing paths between different places.

Each edge (*i*, *j*) ∈ *E* is associated with three parameters: the travel cost *c*_*ij*_, the travel distance *d*_*ij*_, and the travel time *t*_*ij*_ between the two places *i* and *j*.

The objective is to find the optimal itinerary *I*, which is a sequence of places, visits each place exactly once, and minimizes the total travel cost *C(I)*, distance *D(I)*, and time *T(I)*.

A decision variable *x*_*ij*_ can be defined as a binary variable which is equal to 1 if the itinerary includes the edge (*i*, *j*), and 0 otherwise.

The objectives are:

(1) \begin{eqnarray*}\text{minimize}~C \left( I \right) =\sum _{(i,j)\in E}{c}_{ij}{x}_{ij};\end{eqnarray*}

(2) \begin{eqnarray*}\text{minimize}~D \left( I \right) =\sum _{(i,j)\in E}{d}_{ij}{x}_{ij};\end{eqnarray*}

(3) \begin{eqnarray*}\text{minimize}~T \left( I \right) =\sum _{(i,j)\in E}{t}_{ij}{x}_{ij}.\end{eqnarray*}

subject to:

• Each place in the itinerary *I* is left only once:

(4) \begin{eqnarray*}\sum _{j=1}^{n}{x}_{ij}=1,\forall i\in P;\end{eqnarray*}

• Each place in the itinerary *I* is reached only once:

(5) \begin{eqnarray*}\sum _{i=1}^{n}{x}_{ij}=1,\forall j\in P;\end{eqnarray*}

• Preventing non visited places:

(6) \begin{eqnarray*}\sum _{i\in Q}\sum _{j\in Q}{x}_{ij}\leq \left\vert Q \right\vert -1,\forall Q\subset P,2\leq \left\vert Q \right\vert \leq n-1;\end{eqnarray*}

(7) \begin{eqnarray*}{x}_{ij}\in \left\{ 0,1 \right\} ,\forall i,j\in P.\end{eqnarray*}

This problem is a multi-objective one, which takes into consideration all three objective functions (1–3) simultaneously to identify the optimal solution. Consequently, these functions are consolidated into a single objective function, referred to as the fitness function, as defined in Eq. (11). Constraints (4) and (5) ensure that each place is visited once, while constraint (6) ensures that all places are visited in itinerary *I* (Hao et al., 2023).

## Proposed methodology

**Figure 1:**
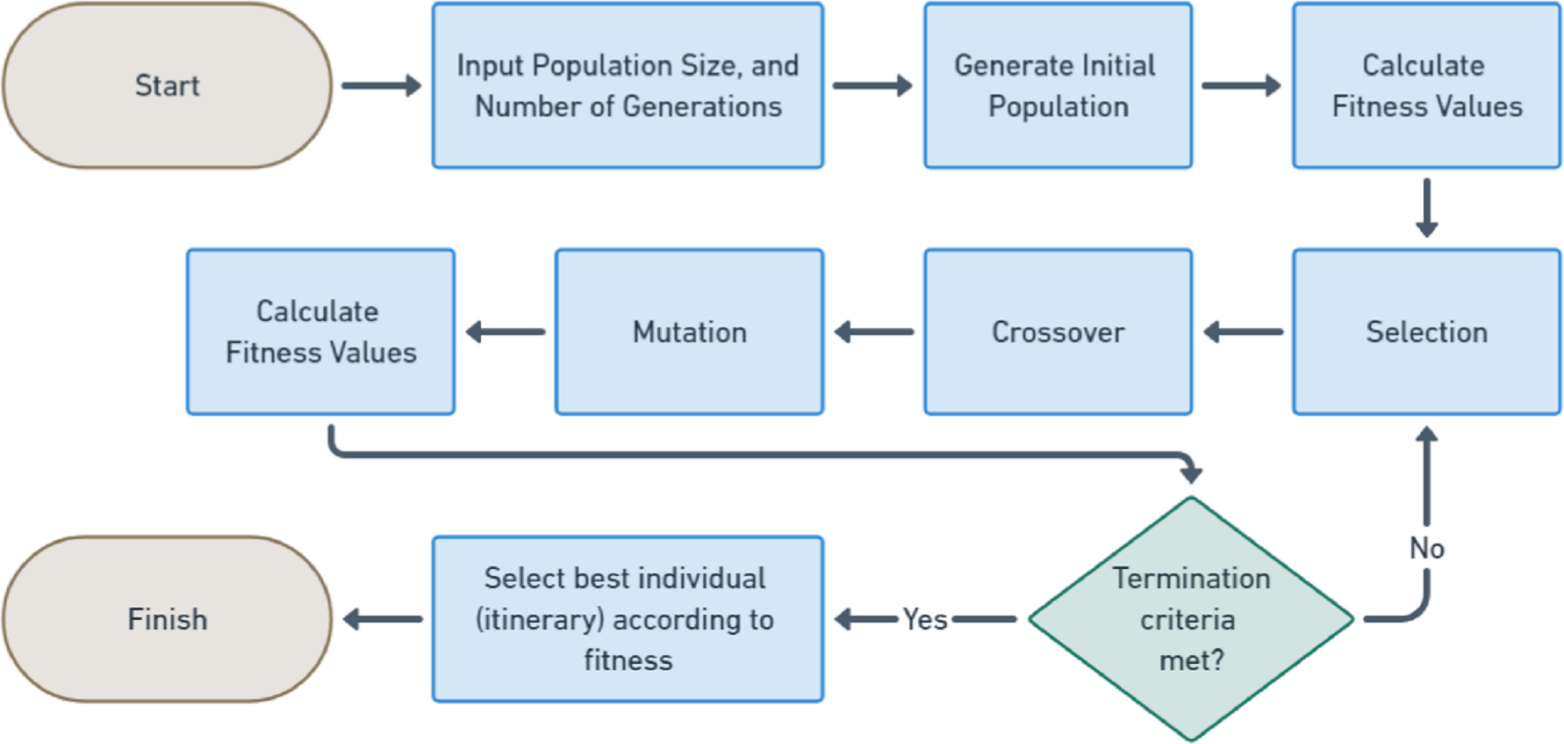
GA flowchart.

This research has important contributions to the field of itinerary recommendation using GA, as it is shown in Fig. 1, which describes the GA process steps conducted in this research. The first contribution is specific to the topic, which is to devise a fitness function that effectively considered multiple factors such as distance, time, and cost associated with different itineraries, as well as the constraints imposed by the tourists’ budget and their preferences for specific activities and attractions. This can contribute to both cost reduction and improved safety outcomes for tourists.

 By minimizing travel distances, these methods not only reduce travel expenses but also potentially decrease the risk of accidents associated with longer journeys. This, in turn, can contribute to a safer and healthier travel experience for tourists. The other contributions are several modifications to improve the performance of the GA, which are applicable in any kind of similar optimization problems in many fields as it is detailed in the Introduction Section. The first modification is for the Tournament Selection method, allowing more efficient and effective selection of individuals for reproduction. The second modification is for the Crossover method, exhibiting superior performance compared to traditional crossover techniques. Final modification is for the swap mutation method, utilizing more dynamic and efficient mutations for next generations.

To assess the effectiveness of these modifications, we conducted extensive experimentation, comparing results across various scenarios. We systematically varied the GA parameters, selection methods, crossover techniques, mutation strategies, and execution time to identify the optimal scenario with the most suitable settings. By undertaking these comparative analyses, we aimed to identify the most efficient and effective configuration of the GA for solving the tourism problem.

Through a comprehensive evaluation of multiple scenarios, we sought to provide insights into the most optimal approach for generating high-quality solutions while considering factors such as computational efficiency and parameter selection.

The key metrics employed in this study are outlined in Table 1. Notably, certain metrics such as travel distance, time, and cost may be construed either as parameters within a broader composite metric, such as fitness, or as standalone metrics, contingent upon the preferences of the tourist, where, for instance, travel distance alone might suffice in cases where considerations of time or costs are of lesser concern.

### Setting-up information matrices

For setting-up real data values of the distances, costs, and travel time spent between different places in both directions, we created their matrices in Excel using visual basic (VB) code and Bing Maps API for 98 different destinations p1, p2, …, p98 in Istanbul.

**Figure 2:**
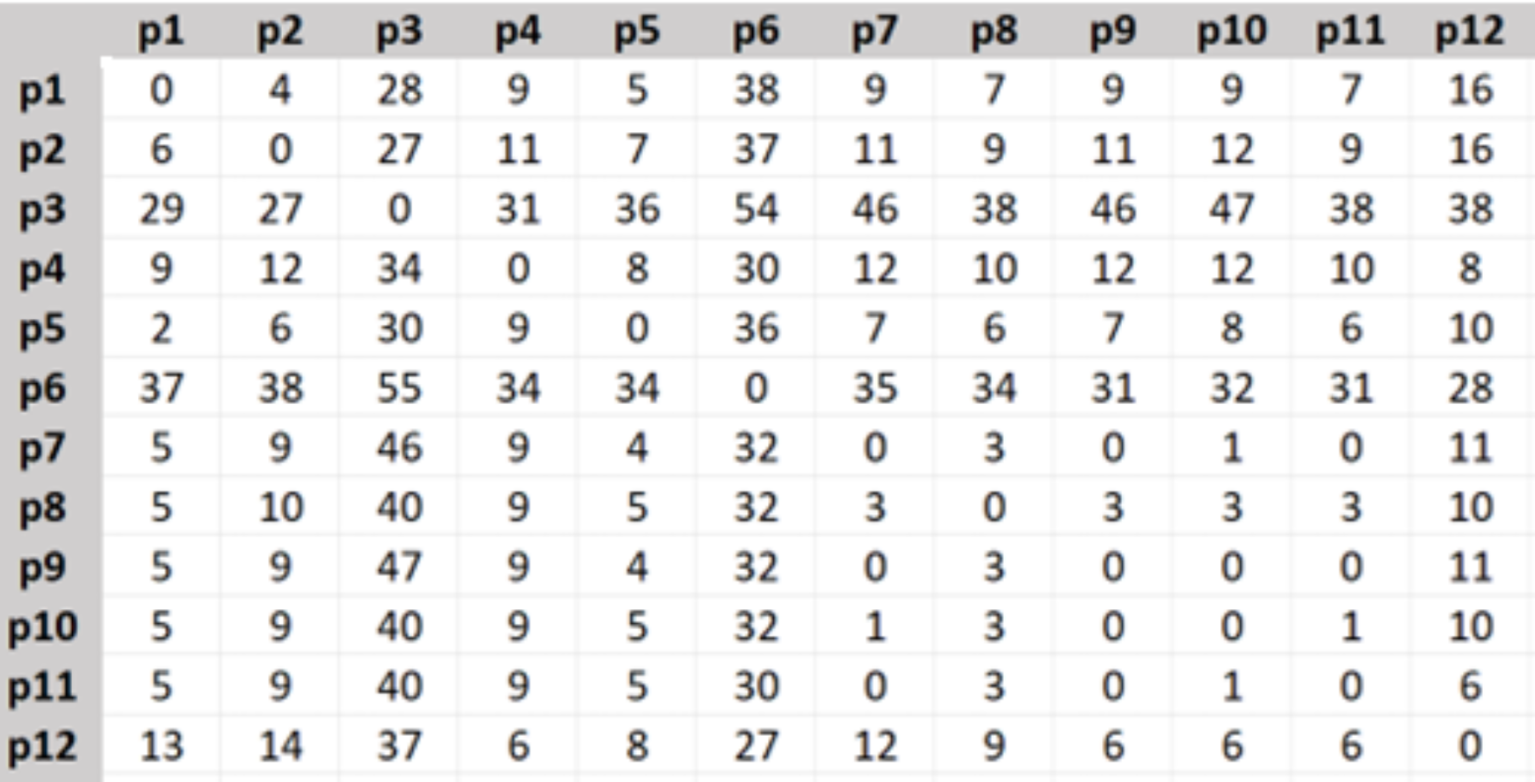
Travel distance matrix.

The travel distance matrix was created as in Fig. 2, the shown numbers are in kilometers.

**Figure 3:**
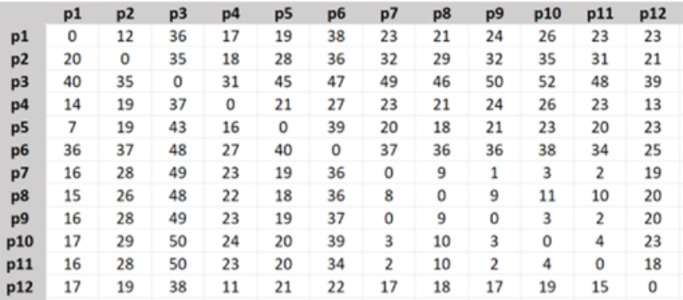
Travel time matrix.

The travel time matrix was created as Fig. 3, the shown numbers are in minutes.

The travel costs *C* were calculated regarding the distances *D* and taking a taxi between each two different places in the following way (IBB; Tuhim, 2023):

1. *D* ≤ 1 km. It is a walking distance and there is no need for a taxi and the cost is zero:

(8) \begin{eqnarray*}C=0;\end{eqnarray*}

2. *D* > 1 km, then:

(9) \begin{eqnarray*}C=aD+b,\end{eqnarray*}

where *a* is the fee charged per kilometer (*e.g.*, 8.51 TL/km), and *b* is the opening fee (*e.g.*, 12.65 TL). It is important to note that the values of *a* and *b* are subject to change based on official regulations;

3. *C* <minimum fee threshold, then:

(10) \begin{eqnarray*}C=\text{minimum fee},\end{eqnarray*}

where the minimum fee is currently set at 40 TL and is periodically updated based on official regulations as well.

**Figure 4:**
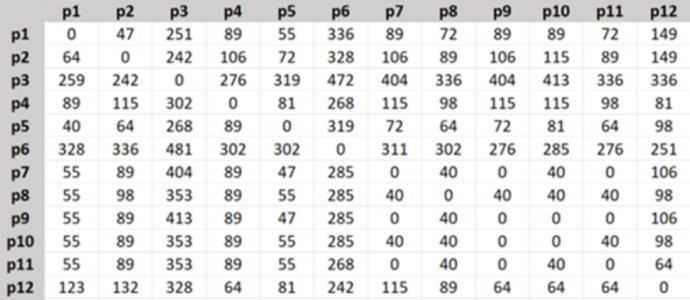
Travel cost matrix.

Then the travel cost matrix was created as in Fig. 4, the shown numbers are in Turkish Lira.

### Creating initial population

**Figure 5:**
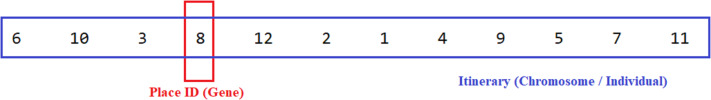
Chromosome structure.

We started creating the initial population by generating random integer numbers from the indices list of the designated places to be visited. For instance, in our case study, we considered a tourist planning to visit 12 museums in Istanbul. Consequently, random numbers ranging from 1 to 12 were generated for each individual in the population without any duplication for the same number in the individual, that the tourist will visit each place once in the itinerary, as it is shown in Fig. 5.

This process was repeated to create an initial population size equivalent to the desired number of itineraries. Like the other researchers, we noticed that the population size must be big enough to increase the probability of getting good solutions with higher fitness values (Piszcz & Soule, 2006), therefore for the 12 places we determined the population size as 100 or 50. However, when it had been less than 50, *e.g.*, 25, we got bad results. However, the utilization of GA significantly aids in the reduction of the search space from 12! (approximately 500 million possible solution) to a mere 100 or 50 itineraries. This substantial reduction in the search space enables the attainment of the optimal solution far more expeditiously compared to conventional methods.

### Devised fitness function

To optimize the fitness function *F*, our objective was to minimize both the total traveled distance *D* and time *T*, as well as the costs *C* associated with the itinerary *I*, ensuring they remained within the predetermined budget *B*. To achieve this, the proposed fitness value for each itinerary can be calculated using Eq. (11):

(11) \begin{eqnarray*}F \left( I \right) = \frac{B-C}{D+T} .\end{eqnarray*}

Minimizing (travel distance, time, and costs) will maximize the devised fitness function. Then we can replace the last 3 objective functions defined in Eqs. (1), (2), (3) by the fitness function Given that the fitness factors encompass distinct natures and units, it is imperative to normalize them before amalgamating the various factors in order to equate the influence of all objectives. Failure to do so would result in a biased solution, compromising the accurate attainment of the optimal solution. In order to mitigate this issue, we employed the range normalization technique (Zaki, Meira Jr & Meira, 2014):

(12) \begin{eqnarray*}Norm \left( X \right) = \frac{X-\min \nolimits \left( X \left( : \right) \right) }{\max \nolimits \left( X \left( : \right) \right) -\min \nolimits \left( X \left( : \right) \right) } ,\end{eqnarray*}

where *X* is the list of values of one parameter of the fitness function.

In our research, all of the fitness function parameters have been normalized within the range of 0 to 1 using Eq. (12). The expense of traveling from point A to point B can range from 0 TL (for nearby locations) to about 500 TL (for distant ones). Similarly, the distance between A and B ranges from 0 km to about 90 km, while the travel time spans from 0 min to 60 min. These parameters exhibit distinct natures and are measured in different units. By utilizing Eq. (12) for normalization, all parameter values can be scaled to a common range of 0 to 1. Consequently, they can be collectively employed in Eq. (11) to determine the fitness of a solution, ensuring that no single parameter dominates over others.

Through the experimental results presented in the Experimental Results section, it becomes evident that certain parameters may experience temporary fluctuations. While the value of one operator decreases, the values of other parameters may momentarily increase, only to eventually return to their decreasing trends. Throughout the GA process, the fitness function exhibits either an increasing trend or remains constant, without any instances of decreasing values, as consistently depicted in all fitness Figures.

### Fitness function and itineraries quality

To assess the efficacy of the proposed fitness function, an evaluation of individuals was conducted, classifying them into three distinct groups based on their entropy values: low diversity, high diversity, and medium diversity. This classification was performed after clustering the places into their respective neighborhoods, ensuring that places within the same neighborhood were grouped together. Subsequently, the individuals’ sequences were modified, replacing the places with their corresponding neighborhood clusters. The entropy *H* of each individual *x* was quantified using Eq. (13):

(13) \begin{eqnarray*}H \left( x \right) =-\sum _{i}{P}_{i}\cdot \log \nolimits 2 \left( {P}_{i} \right) ,\end{eqnarray*}

where:

• *x* is an individual which represents an itinerary in our problem.
• *i* = 1, …, *n* is the index of element in the individual.
• *n* is the number of places in the itinerary.
• *P*_*i*_ is the probability of a particular element (set) occuring in the individual.

Entropy serves as a measure of an individual’s diversity level, where a higher entropy value signifies a greater diversity of places within the individuals’ sequences. Conversely, a lower entropy value indicates a lower diversity, implying that the places from the same neighborhood appear consecutively in the itinerary. In contrast, individuals characterized by high diversity exhibit a pattern wherein most neighboring places do not appear sequentially in the itinerary sequences (Spellerberg & Fedor, 2003).

**Figure 6:**
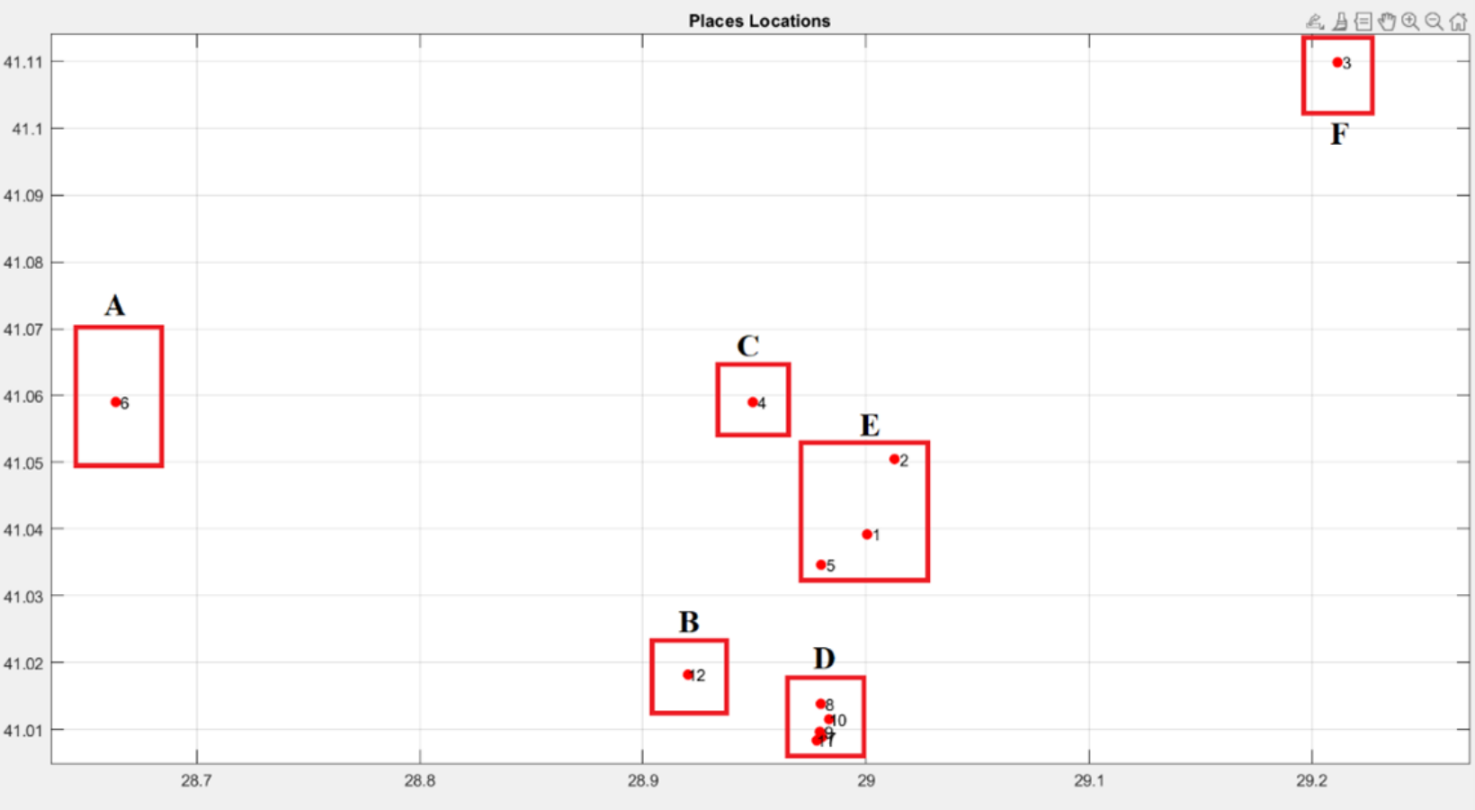
Places locations and clusters.

Example: Let us cluster 12 different places according to their neighborhoods into 6 separated clusters A,B,C,D, and E as Fig. 6 and Table 2.

As evident from Fig. 6, when devising an itinerary encompassing the 12 designated places, it is imperative for the tourist not to leave any neighborhood before visiting all its specified locations.

Consequently, to achieve this constraint, the individual’s diversity should be minimized, thereby resulting in the lowest possible value for the individual entropy defined in Eq. (13).

Now let’s compare the entropy and the fitness values of some samples of (high, low, and medium) diversity individuals as it is seen in Table 3.

**Figure 7:**
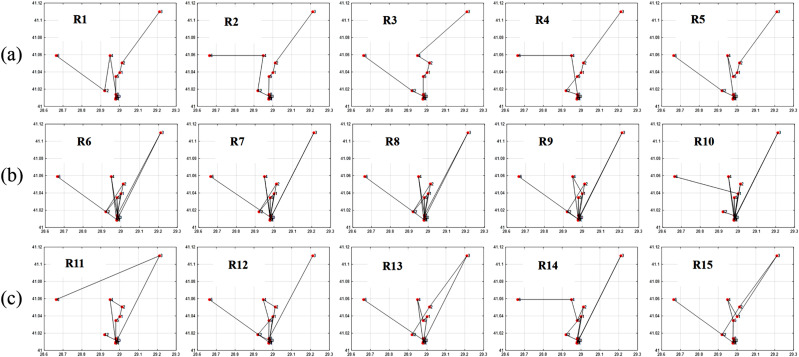
Itineraries graphs of Table 3 individuals. (A) Best itineraries with high fitness and low entropy; (B) worst itineraries with low fitness and high entropy; (C) moderate itineraries with medium fitness and medium entropy.

**Figure 8:**
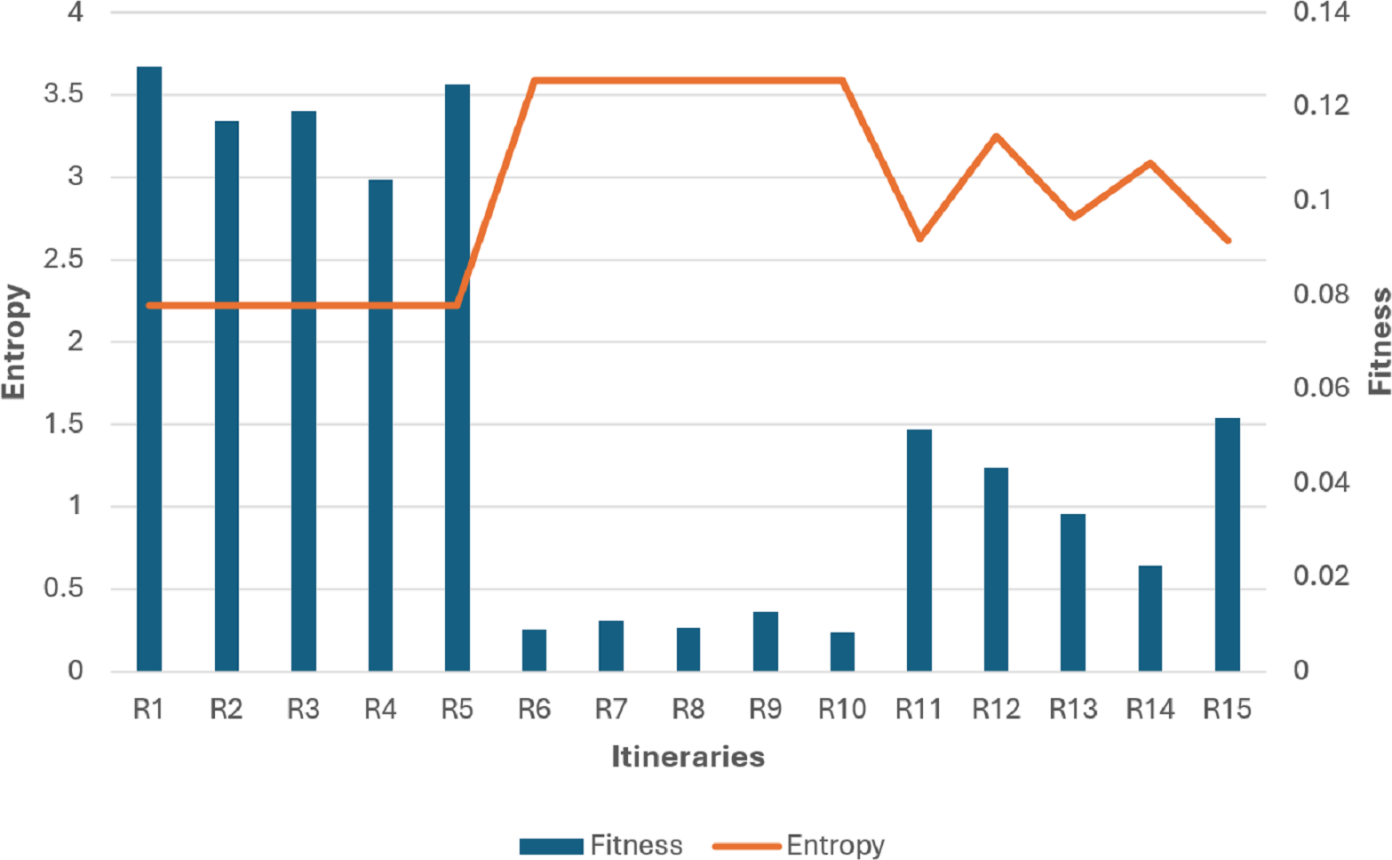
Fitness and entropy relationship graph of Table 3 individuals.

The findings depicted in Figs. 7 and 8 demonstrate a significant relationship between individual diversity and fitness levels. Specifically, low-diversity individuals exhibit high fitness values and best itineraries, while high-diversity individuals display lower fitness values and worst itineraries.

Additionally, medium diversity individuals exhibit fitness values not good and not bad with their moderate itineraries.

Now let’s classify the itineraries to optimal and non-optimal according to their fitness values *F* and entropy *H*, as in Table 4.

1. (Low *F* & Low *H*): It is non-optimal itinerary because the order of places is not optimal even though the diversity is low, like: A D D D D D F B E E E C (taking into consideration that the places in the same neighborhood are close enough to each other in a way that the order in the same group doesn’t have that great effect on the fitness)
2. (Low *F* & High *H*): It is non-optimal itinerary because of leaving the neighborhood without visiting all its places which affected the fitness parameters values. Like: R6, R7, R8, R9, and R10 in Fig. 8.
3. (High *F* & Low *H*): It is optimal itinerary whereas all of places in the same neighborhood are visited before leaving it, and at the same time the order of places in the itinerary is optimal. Like: R1, R2, R3, R4, and R5 in Fig. 8.
4. (High *F* & High *H*): It is impossible case for our dataset that it may occur only when each group includes just one place inside it. In our example, D and E groups include five and three places, respectively.

From the cases in Table 4, we conclude that for our example dataset, it is enough to consider the itineraries with high fitness to be optimal that the high fitness itinerary must has low entropy, but the opposite case is not necessarily to be correct, that the low entropy itinerary may has high or low fitness value. Therefore, The R1, R2, R3, R4, and R5 patterns will be considered as optimal. And the rest are non-optimal itineraries.

**Table 2:** Places clusters.

**Cluster**	**Places**
A	6
B	12
C	4
D	7, 8, 9, 10, 11
E	1, 2, 5
F	3

**Table 3:** Fitness and entropy values of different individuals.

**Individual**	**Individual sequences**	**Fitness/** **entropy**	**Diversity**
R1	6, 12, 4, 10, 9, 7, 11, 8, 5, 1, 2, 3	0.1286	Low
	A, B, C, D, D, D, D, D, E, E, E, F	2.2213	
R2	6, 4, 12, 10, 9, 8, 7, 11, 5, 1, 2, 3	0.1168	Low
	A, C, B, D, D, D, D, D, E, E, E, F	2.2213	
R3	6, 12, 10, 9, 7, 11, 8, 5, 1, 2, 4, 3	0.1189	Low
	A, B, D, D, D, D, D, E, E, E, C, F	2.2213	
R4	6, 4, 10, 9, 7, 11, 8, 12, 5, 1, 2, 3	0.1044	Low
	A, C, D, D, D, D, D, B, E, E, E, F	2.2213	
R5	6, 12, 10, 9, 7, 11, 8, 4, 5, 1, 2, 3	0.1248	Low
	A, B, D, D, D, D, D, C, E, E, E, F	2.2213	
R6	6, 7, 3, 8, 2, 12, 1, 9, 5, 10, 4, 11	0.0089	High
	A, D, F, D, E, B, E, D, E, D, C, D	3.5850	
R7	6, 10, 3, 9, 2, 12, 1, 11, 5, 7, 4, 8	0.0107	High
	A, D, F, D, E, B, E, D, E, D, C, D	3.5850	
R8	6, 7, 5, 9, 2, 12, 1, 8, 3, 10, 4, 11	0.0093	High
	A, D, E, D, E, B, E, D, F, D, C, D	3.5850	
R9	6, 9, 12, 2, 10, 3, 7, 4, 1, 11, 5, 8	0.0127	High
	A, D, B, E, D, F, D, C, E, D, E, D	3.5850	
R10	6, 1, 9, 3, 7, 4, 10, 2, 11, 5, 8, 12	0.0082	High
	A, E, D, F, D, C, D, E, D, E, D, B	3.5850	
R11	6, 3, 11, 7, 8, 5, 1, 2, 4, 9, 10, 12	0.0513	Medium
	A, F, D, D, D, E, E, E, C, D, D, B	2.6258	
R12	6, 9, 2, 4, 5, 11, 10, 12, 1, 7, 8, 3	0.0434	Medium
	A, D, E, C, E, D, D, B, E, D, D, F	3.2516	
R13	6, 10, 11, 8, 9, 4, 5, 1, 7, 3, 2, 12	0.0333	Medium
	A, D, D, D, D, C, E, E, D, F, E, B	2.7516	
R14	6, 4, 5, 2, 9, 7, 3, 11, 10, 12, 1, 8	0.0225	Medium
	A, C, E, E, D, D, F, D, D, B, E, D	3.0850	
R15	6, 7, 8, 9, 10, 11, 5, 4, 1, 3, 2, 12	0.0539	Medium
	A, D, D, D, D, D, E, C, E, F, E, B	2.6175	

This compelling evidence validates the efficacy of our proposed fitness function, indicating its suitability for effectively evaluating any individual within the GA process.

### Modification of the selection method

We applied the random selection method, then the tournament selection method in the parents selection step in GA. As displayed in the Experimental Results section, the experimental results indicate the superiority of the tournament selection method over random selection. However, as we monitored the individuals across generations, it became evident that the tournament selection method led to an increase in the number of identical individuals within each generation. This phenomenon occurred due to the presence of elitism (Das & Panigrahi, 2009), which ensured that the best solutions remained prominent in subsequent generations, and the efficacy of crossovers, gradually generating offspring with improved orderings.

To address the issue of duplicated individuals within the tournament group, we modified the tournament selection method. Specifically, if all individuals within the tournament group were identical, we selected the other different parent from outside the tournament group with the highest fitness in the population. This modification yielded positive outcomes. However, as the generations progressed, we observed that the individuals once again became congruent, rendering the selection of congruent parents a time-consuming process.

To overcome this limitation, as it is explained in detail in the Tuning Swap Mutation section, after reaching the stage of high congruence within the generation, we increased the mutation rate from 0.1 to 0.9 to increase the diversity level in the population. By introducing more mutations and generating additional crossovers, we enhanced the probability of obtaining superior offspring compared to previous iterations, leading to improved solutions. Throughout the empirical evaluation, we found that in the GA scenarios with the normal tournament selection method, the high congruence in population causes GA to converge so quickly to suboptimal solutions.

**Table 4:** Classifying itineraries based on their fitness (F) and entropy (H).

**Itinerary (R)**		The extent of visiting all places in the same neighborhood before leaving it in the itinerary.	
		**Low (** ***H***_*R*_ **)**	**High (** ***H***_*R*_ **)**
The extent of minimizing travel distance, cost, and time for the itinerary.	**Low (** ***F***_*R*_ **)**	1. Non-optimal itinerary	2. Non-optimal itinerary
	**High (** ***F***_*R*_ **)**	3. Optimal itinerary	4. Impossible

While in the GA scenarios with the modified tournament selection method, with less congruent individuals in the population we could obtain superior offspring with higher fitness values, of course with more iterations, as it is seen in the experimental results. This adjustment effectively addressed the issue of stagnation caused by congruent individuals and facilitated the continued progress of the GA toward optimal solutions. The Pseudocode of utilized selection methods is given in Algorithm 1 .


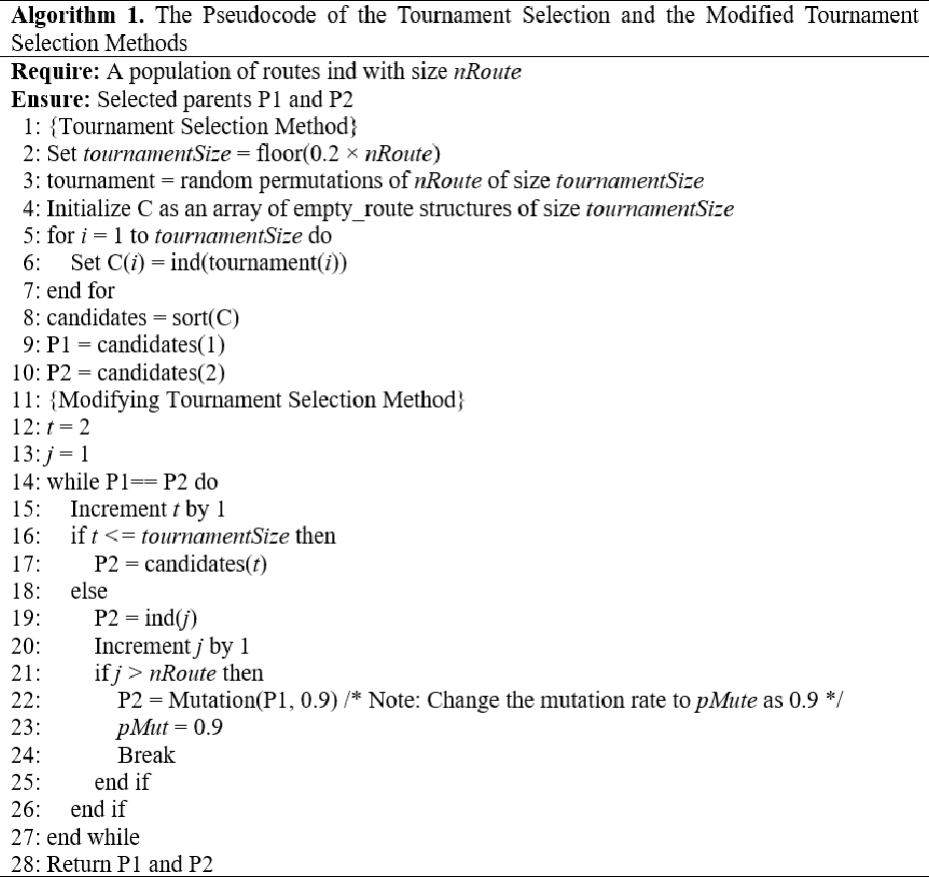


### Crossover step

**Figure 9:**
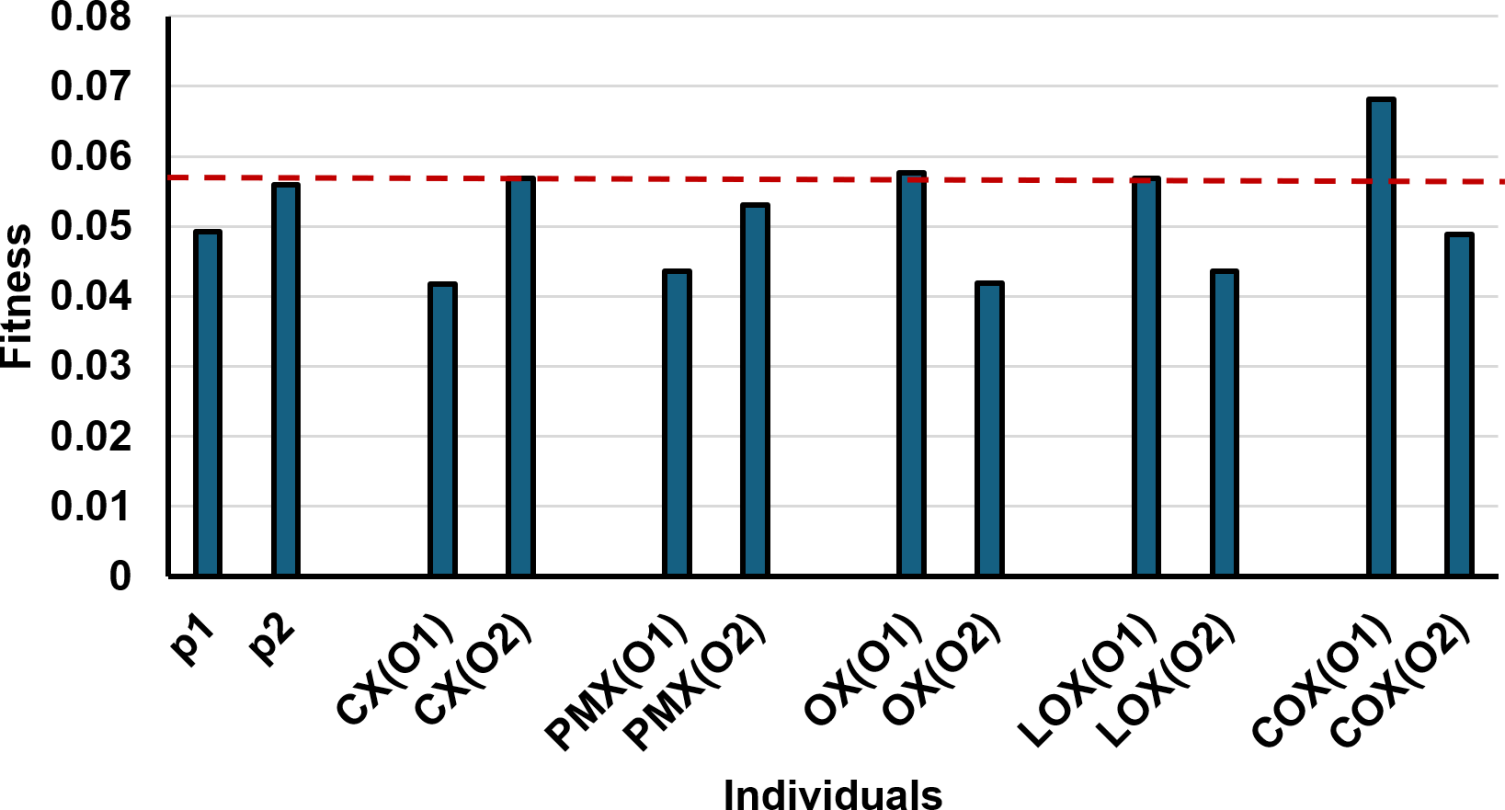
Fitness values of parents and offsprings of different crossover methods for the given example.

Following the selection step, we initially utilized the CX crossover method. We selected it as a non-order-based crossover approach to compare it with our proposed order-based COX crossover method and evaluate its efficiency. After making some initial comparison with the other already exist crossover methods we got the following results as it is seen in Table 5 and Fig. 9.

**Table 5:** Description of the crossover methods with an example.

**Crossover** **method**	**Itineraries**	**Fitness**	**Description**
	Parent1: [1 7 2 — 4 3 — 6 5]	P1 = 0.0492	
	Parent2: [1 5 3 — 2 6 — 4 7]	P2 = 0.0559	
CX	Offspring1: [1 7 3 2 6 4 5] Offspring2: [1 5 2 4 3 6 7]	CX(O1) = 0.0417 **CX(O2) = 0.0569**	The first position (1) remains unchanged in the offspring sequences. The cycle positions 7,5,7 are also retained in their positions. However, the other positions 2, 4, 3, and 6 are swapped with 3, 2, 6, and 4, respectively, in the offspring.
PMX	Offspring1: [1 7 4 — 2 6 — 3 5] Offspring2: [1 5 6 — 4 3 — 2 7]	PMX(O1) = 0.0436 PMX(O2) = 0.0531	The middle parts are swapped between parents. The repeated places (2 and 6) are mapped to their corresponding values (4 and 3) in Offspring1. Similarly, in Offspring2, the repeated places (3 and 4) are mapped to (6 and 2) respectively.
OX	Offspring1: [7 4 3 — 2 6 — 5 1] Offspring2: [5 2 6 — 4 3 — 7 1]	**OX(O1) = 0.0577** OX(O2) = 0.0419	The middle parts are swapped, then for the first offspring starting from the second cut point 6, 5, 1, 7, 2, 4, 3 will be 5, 1, 7, 4, 3 after pruning replications. And in the second offspring, starting from the second cut point 4, 7, 1, 5, 3, 2, 6 will be 7, 1, 5, 2, 6.
LOX	Offspring1: [1 5 2 — 4 3 — 6 7] Offspring2: [1 7 4 — 2 6 — 3 5]	**LOX(O1)=0.0569** LOX(O2) = 0.0436	The middle parts are not swapped, the line of places 1, 7, 2, 6, 5 in the first parent will be reordered as its order in second parent to be 1, 5, 2, 6, 7 in the first offspring. In the same way, the line 1, 5, 3, 4, 7 in the second parent will be 1, 7, 4, 3, 5 in the second offspring.
Proposed COX	Offspring1: [1 2 7 — 4 3 — 5 6] Offspring2: [1 3 5 — 2 6 — 7 4]	**COX(O1) = 0.0681** COX(O2) = 0.0488	The middle parts are not swapped, the edges are not represented as one line like LOX, *i.e.*, the two edges of the first parent 1, 7, 2 and 6, 5 will be reordered separately as their orders in the other parent to be 1, 2, 7 and 5, 6 respectively. In a similar way, the second parent edges 1, 5, 3 and 4, 7 will be 1, 3, 5 and 7, 4 respectively in the second offspring.

As depicted in Fig. 9, the offspring produced by our proposed crossover method, COX, exhibit higher fitness values compared to offspring generated by alternative methods and even their respective parents. This indicates that our COX method may outperform all other methods in our problem. While the other crossover methods (CX, PMX, OX, and LOX) have demonstrated effectiveness in various domains, Fig. 9 reveals that their offspring solutions achieve similar fitness values, with some of them like PMX ones not surpassing those of their parents. The good fitness values in Table 5 are formatted in bold and underlined.

However, through continuous monitoring of the CX performance across subsequent generations, we observed that it exhibited slow convergence. Frequently, the CX method produced offsprings with either minimal or no changes from the parent solutions. This slow progress prompted us to propose an alternative and more efficient crossover method, named COX.

Through extensive experimentation and analysis, we discovered that the COX method outperformed the CX method in terms of efficiency and speed, yielding superior results for the metrics mentioned in Table 1. The proposed method offered a solution to the limitations encountered with the CX, enabling more significant changes in the offspring and leading to improved outcomes. The adoption of the COX method addressed the issue of sluggish convergence and provided a more effective approach for achieving desirable results within the GA.

**Figure 10:**
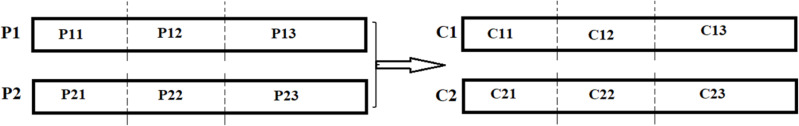
COX diagram.

#### The COX method description

**Figure 11:**
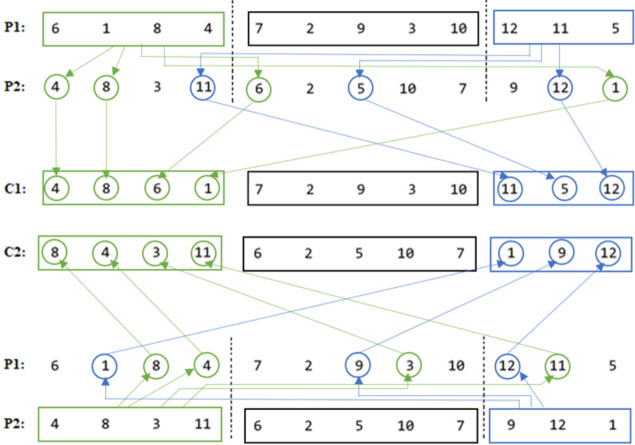
COX descriptive numerical example.

Our proposed COX method diagram is shown in Fig. 10, and its pseudocode is given in Algorithm 2 . Then it is clarified in Fig. 11 using numerical example.


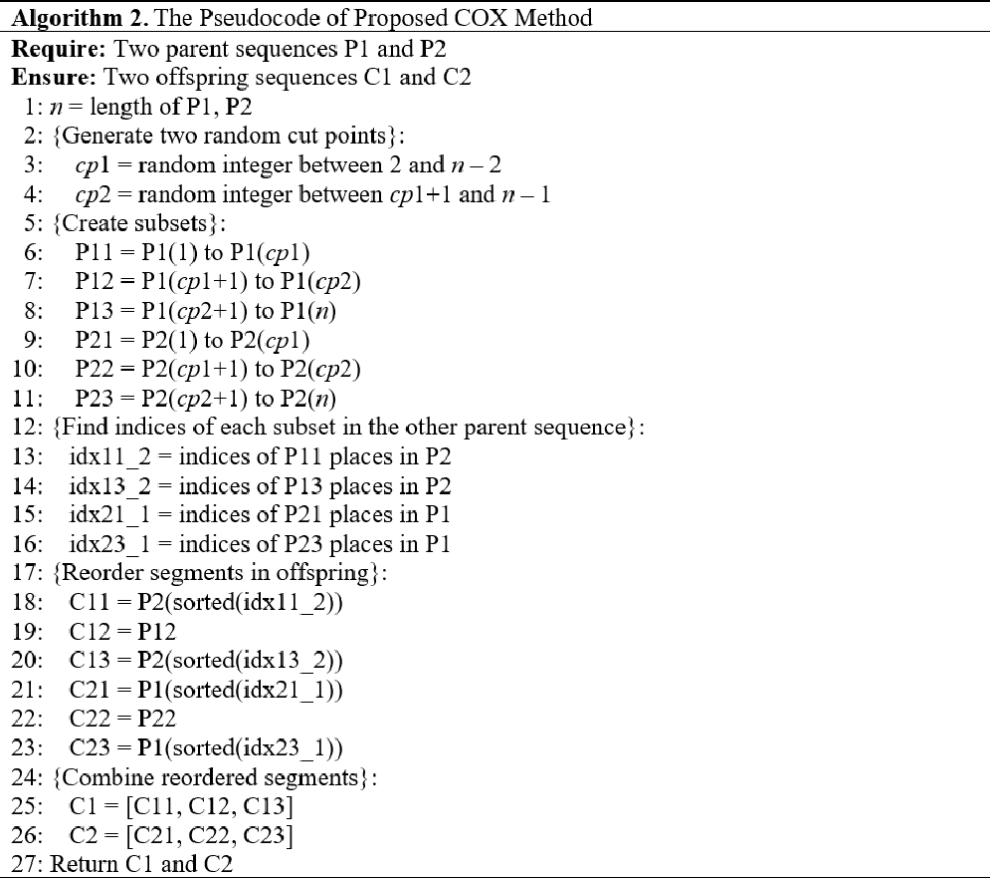


As it is seen in the example in Fig. 11, the middle parts of the parents are not changed in the children, even they were not swapped, in order not to get any duplication problem.

• C12 = P12 = [7 2 9 3 10].
• C22 = P22 = [6 2 5 10 7].
• C11 = Order(P11) in P2 = [4 8 6 1].
• C13 = Order(P13) in P2 = [11 5 12].
• C21 = Order(P21) in P1 = [8 4 3 11].
• C23 = Order(P23) in P1 = [1 9 12].

The utilization of the COX technique in our study ensures the prevention of duplication issues by facilitating the inheritance of specific traits from one parent to each child while incorporating characteristics from the other parent. This methodology effectively promotes the generation of offspring with unique genetic compositions, thereby enhancing the diversity and variability within the population. Notably, unlike other order crossover methods that involve wrapping or filling discrete positions for the offspring, the COX method maintains the first-place position of each individual, preserving the established optimal order within the offspring sequences. As a result, the COX method facilitates both effective exploration and exploitation of the solution space, leading to improved solution quality. Furthermore, it mitigates any concerns related to duplication or deviation from the optimal solution.

### Tuning swap mutation

We optimized and implemented the Swap Mutation method to enable simultaneous swaps between multiple places, rather than being restricted to pairwise swaps while adhering to a specified mutation rate (Chand & Mohanty, 2013; Shahab et al., 2019). Moreover, we employed a dynamic approach in utilizing the mutation operator. Initially, a low mutation rate (0.1) was applied to ensure minimal interference with the convergence toward the optimal solution. However, as the generations progressed and all individuals became identical, a higher mutation rate (0.9) was employed in order to make diversity in the population sooner. This increase in mutation rate introduced significant changes to the subsequent individuals, thereby increasing the probability of obtaining improved solutions. By employing this dynamic mutation strategy, we aimed to facilitate exploration and diversification in the GA, promoting the discovery of superior solutions. As an example, for x = [12, 7, 11, 4, 9, 5, 1, 3, 8, 2, 10, 6]:

• If the Mutation Rate = 0.1 then x = [12, 7, 11, 4, 9, 5, 1, 3, 8, 2, 10, 6] (No change).
• If the Mutation Rate = 0.9 then x = [12, 11, 7, 4, 9, 1, 5, 3, 8, 10, 2, 6] (Many changes).

To introduce a mutation rate to the individual x, which consists of 12 members, we constructed a 12 ×12 matrix of 0’s and 1’s randomly based on the specified mutation rate. For instance, a mutation rate of 0.5 indicates that approximately 50% of the matrix elements will be assigned a value of 1. Consequently, if the 3rd and 7th rows of the matrix are fully populated with 1’s, only the 3rd and 7th elements of individual x will be swapped with the 4th and 8th elements, respectively.

As shown in the given example, a mutation rate of 0.1 was employed, resulting in a 12 ×12 matrix with 10% of its elements assigned a value of 1. The probability of obtaining a fully populated row of 1’s in this matrix is 0.1ˆ12 = 1.0000e−12. As a result, x remains unchanged.

In contrast, with a mutation rate of 0.9, which yields a probability of 0.9ˆ12 = 0.2824, approximately 25–30% of the individuals undergo changes. For instance, the last sequence is derived from x by swapping the 2nd, 6th, and 10th positions with their adjacent 3rd, 7th, and 11th elements, respectively.

## Experimental Results

The experiments were conducted using MATLAB R2022b on a laptop equipped with an AMD Ryzen 5 processor, 16GB RAM, and Windows 10 operating system.

During the development of the GA MATLAB code, the utilization of structures proved essential in efficiently organizing and storing crucial information and parameters related to the problem at hand. Notably, the “inputs” struct was employed to encapsulate the various tourist constraints, including budgetary considerations and the specific destinations to be visited. Additionally, the “parameters” struct was implemented to encompass the essential parameters utilized within the GA function. These parameters encompassed factors such as the number of generations or iterations of the algorithm, the quantity of individuals within each generation referred as (population size) representing the algorithm’s generated number of itineraries or solutions, the mutation rate, and other relevant parameters essential to the GA execution. The use of structures within the code provided a cohesive framework for managing and manipulating the pertinent information and parameters of the problem.

This study utilizes two datasets for itinerary recommendation. The first dataset, compiled specifically for this research, contains information on the 12 most visited museums in Istanbul. The selection of these museums was informed by the studies of Cevdet Altunel & Erkut (2015) and Cetin & Bilgihan, (2016). This dataset, along with additional information matrices containing travel distances, costs, and durations, are provided as supplemental files in (.mat) format for efficient processing by the GA. The second dataset is the well-established Burma benchmark dataset retrieved from TSPLIB. This dataset is also provided as a supplemental file in (.mat) format. Notably, converting all datasets and information matrices to (.mat) files significantly accelerated GA execution time, reducing it from over 50 min to under 20 s.

**Figure 12:**
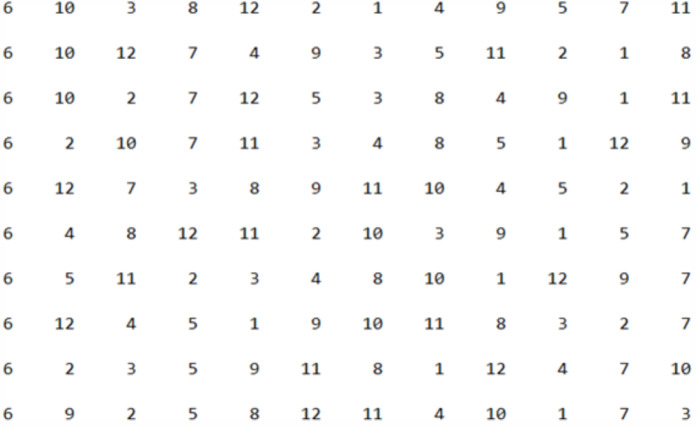
The first 10 individuals in the initial population.

Starting with applying GA on Istanbul museums dataset, to construct the initial population, we opted to begin the itinerary from the 6th place, specifically the “Pelit Chocolate Museum”. The initial population was randomly generated from the 12 museums, and Fig. 12 presented below illustrates the first 10 itineraries within the initial population.

To facilitate a comprehensive understanding of the results and assess the efficacy of the proposed and developed methods, we established 12 distinct scenarios for GA setting. Table 6 presented below outlines the various configurations employed during the execution of the GA. It is important to note that all scenarios utilized the same initial population.

**Table 6:** Different scenarios for GA setting.

**Scenarios**	**Number of generations**	**Population Size**	**Selection method**	**Crossover method**
S1	100	100	Random	CX
S2	100	100	Tournament	CX
S3	100	100	Modified tournament	CX
S4	100	100	Random	COX
S5	100	100	Tournament	COX
S6	100	100	Modified tournament	COX
S7	50	50	Random	CX
S8	50	50	Tournament	CX
S9	50	50	Modified tournament	CX
S10	50	50	Random	COX
S11	50	50	Tournament	COX
S12	50	50	Modified tournament	COX

**Figure 13:**
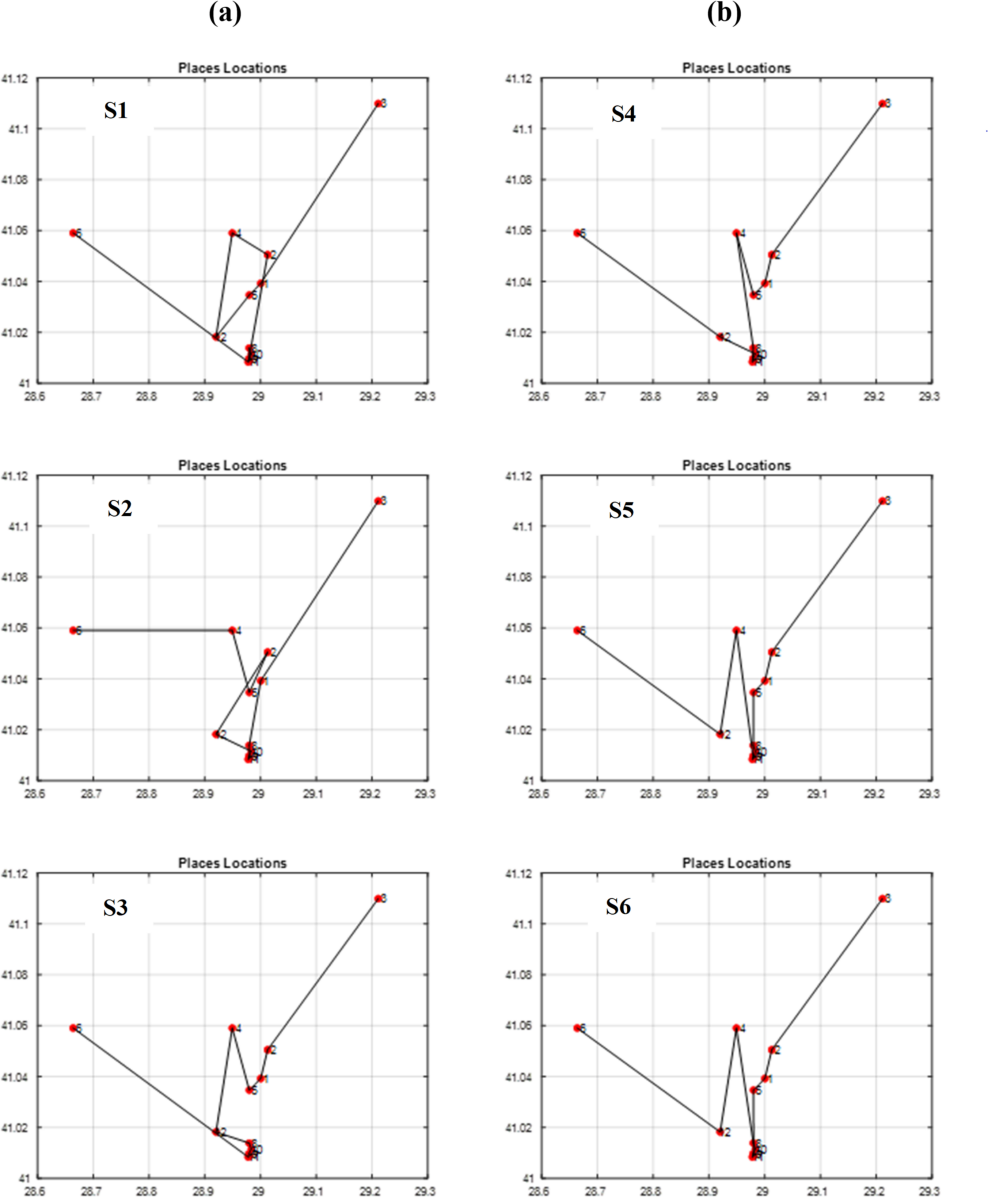
The best itineraries graphs of scenarios S1 through S6. (A) GA Scenarios with CX crossover; (B) GA Scenarios with COX crossover.

**Figure 14:**
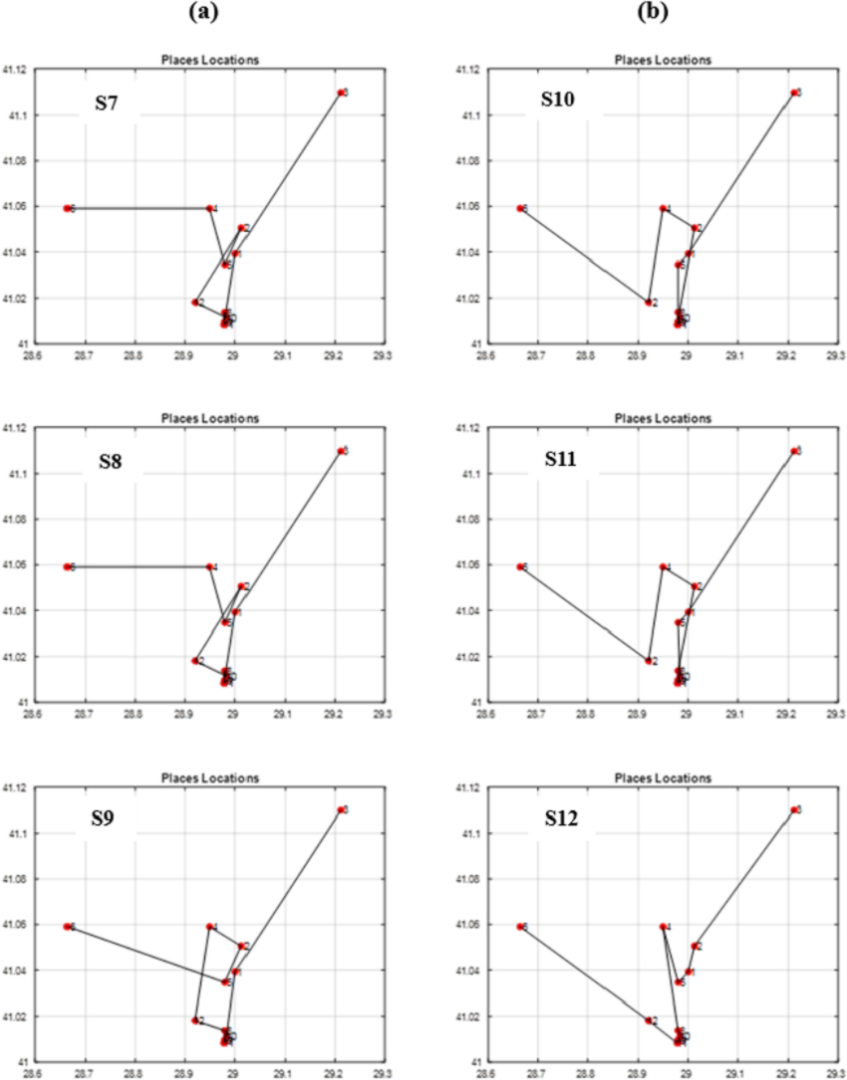
The best itineraries graphs of scenarios S7 through S12. (A) GA Scenarios with CX crossover; (B) GA Scenarios with COX crossover.

By running the GA scenarios given in Table 6, we obtained the following outcomes in Table 7 and the following graphs in Figs. 13 and 14. The figure of each scenario displays the optimal itinerary path, representing the best solution achieved in that particular scenario.

**Table 7:** Istanbul GA results.

**Scenario**	**Best fitness**	**First generation number which reached best fitness**	**GA run time** ** (Sec)**
S1	0.0893	6th	17.1621
S2	0.0942	3rd	16.7899
S3	0.1195	24th	18.6036
S4	0.1251	12th	17.3668
S5	0.1238	4th	18.3003
S6	0.1339	7th	19.4983
S7	0.0870	7th	7.5322
S8	0.0870	2nd	7.3873
S9	0.0959	10th	6.9225
S10	0.1089	13th	6.8489
S11	0.1139	5th	7.7401
S12	0.1267	18th	7.9080

To evaluate the effectiveness of our proposed approach, we compared the fitness values of scenarios using our proposed approach (S6 and S12) to those employing the traditional approach (S2 and S8) in Table 7. This comparison focuses on both selection and crossover operators, providing a comprehensive assessment of the overall enhancement achieved. The results demonstrate a significant improvement in fitness with the proposed approach. Scenario S6 outperforms S2 by 42.14%, and S12 exhibits a 45.63% increase in fitness compared to S8. By calculating the average of these enhancements, we can determine an overall enhancement rate of 43.89% in the GA when using our proposed method.

Through vertical comparisons of the scenarios in Fig. 13, we observed that the second row outperforms the first row, indicating that the S2 and S5 itineraries are superior to S1 and S4 respectively, exhibiting earlier stability in fitness, as it is seen in Table 7. Consequently, we can conclude that the “Tournament” selection method outperforms the “Random” selection method.

Other observations found that the third row significantly surpasses the second row in terms of itinerary quality, with the S3 and S6 itineraries and fitness values being superior to those of S2 and S5 respectively. Consequently, we conclude that the “Modified Tournament” selection method is superior to the “Tournament” selection method.

It can also be noticed by conducting horizontal comparisons that the second column is vastly superior to the first column. Specifically, itineraries S4, S5, and S6 are superior to S1, S2, and S3 respectively. Therefore, we can conclude that the suggested COX method is more efficient, superior, and faster than the CX method.

**Figure 15:**
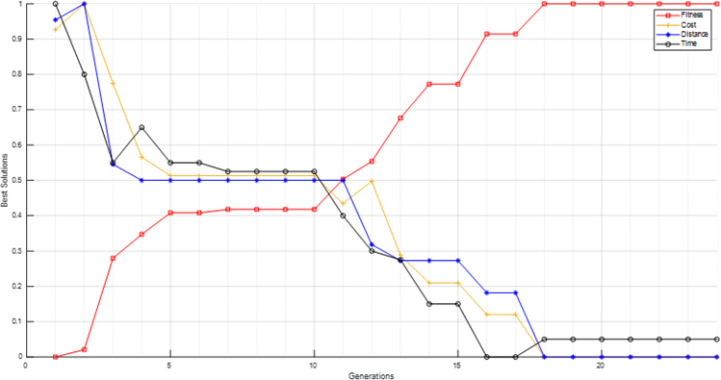
S12 scenario fitness and its parameters values for best solutions in each generation.

Through conducting both vertical and horizontal comparisons on Fig. 14, we have reached similar conclusions to those derived from Fig. 13 and Table 7. These findings confirm the superiority of the suggested and modified methods over the other approaches employed in previous studies. While scrutinizing all of the scenarios fitness graphs, we gained comprehensive insights into the optimality of the optimal solutions within each scenario. Those trends closely resemble the patterns observed in S6 and S12 fitness and other parameters graphs depicted in Fig. 15 for the S12, as corroborated by the data presented in Table 8.

**Table 8:** Performance analysis of distance, time, cost, and fitness throughout S12 scenario generations.

**Generation step**	**Distance**	**Time**	**Cost**	**Fitness**
1–2	Inc.	Dec.	Inc.	Inc.
2–3	Dec.	Dec.	Dec.	Inc.
3–4	Dec.	Inc.	Dec.	Inc.
4–5	Const.	Dec.	Dec.	Inc.
5–6	Const.	Const.	Const.	Const.
6–7	Const.	Dec.	Const.	Inc.
7–10	Const.	Const.	Const.	Const.
10–11	Const.	Dec.	Dec.	Inc.
11–12	Dec.	Dec.	Inc.	Inc.
12–13	Dec.	Dec.	Dec.	Inc.
13–14	Const.	Dec.	Dec.	Inc.
14–15	Const.	Const.	Const.	Const.
15–16	Dec.	Inc.	Dec.	Inc.
16–17	Const.	Const.	Const.	Const.
17–18	Dec.	Inc.	Dec.	Inc.
18–50	Const.	Const.	Const.	Const.

Table 8 reflects the essence of optimization efforts, where achieving the optimal solution in itinerary recommendation does not solely entail minimizing distance. Several factors such as time and cost also play pivotal roles in the decision-making process. Despite this, the overarching trend in their respective graphs indicates a downward trajectory. In more detail, the values of the three parameters (distance, time, and cost) exhibit fluctuations, as evidenced by the 2nd generation where both distance and cost values increase, while time sees a decrease. A reversal of this pattern is observed in the 4th, 16th and the 18th generation. In the 3rd and 13th generations all of the parameters decrease alongside. Another pattern, with a constant value for the distance and a decrease in both time and cost, is noticed in the 5th, 11th, and 14th generations. The parameters values remain steady in the 18th generation until the end. This emphasizes the efficacy of the devised fitness function, particularly considering the overarching pattern indicating an ascending trajectory. This trend remains constant unless all other parameters are held constant, at which point the fitness function stabilizes, devoid of any downward inclination.

**Figure 16:**
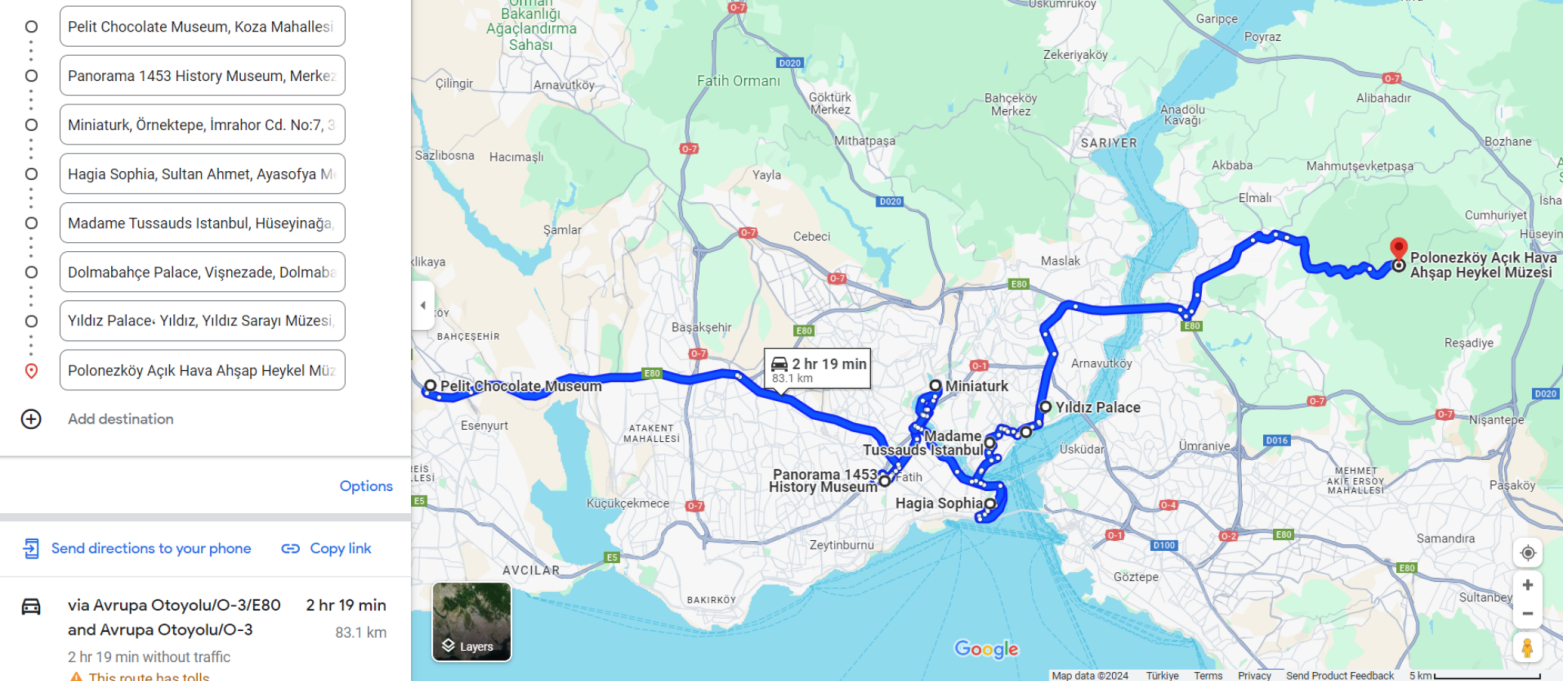
The S6 optimal itinerary on google maps. Map data ©2024 Google.

As it is seen in Table 7, S6 is slightly superior to S12 according to best fitness and reaching it earlier, but S12 is 2.5 times faster than S6. Therefore, the tourist may be supplied with both itineraries S6 and S12 as the most optimal ones. The optimality of the given itinerary may not be immediately evident in the xy-plane; however, when visualized using Google Maps, individuals familiar with the streets of Istanbul can ascertain that it provides the most optimal itinerary in terms of trip distance, cost, and time. Figure 16 displays the Google Maps representation of the optimal itinerary generated in S6, confirming its superiority as the most optimal itinerary.

The itinerary can be displayed on the map by clicking here as well. The produced itinerary’s places by order are:

• (6) Pelit Chocolate Museum.
• (12) Panorama 1,453 History Museum.
• (4) Miniaturk.
• (10–9–7–11–8) Hagia Sophia Neigbourhood.
• (5) Madame Tussauds.
• (1) Dolmabahçe Palace.
• (2) Yildiz Palace Museum.
• (3) Polonezköy Acik Hava Ahşap Heykel Museum.

**Figure 17:**
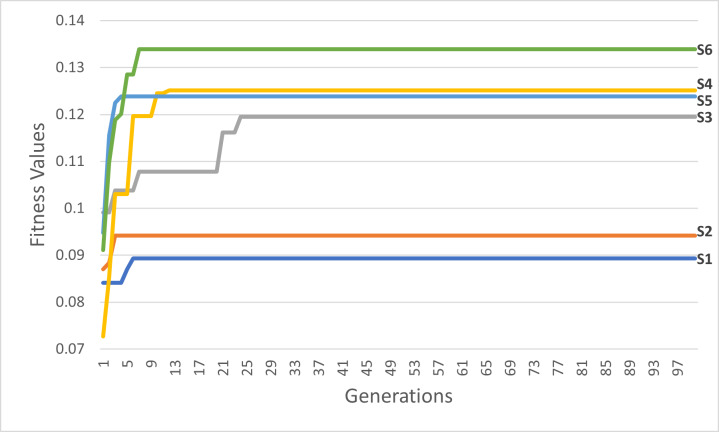
The first six scenarios fitness values.

**Figure 18:**
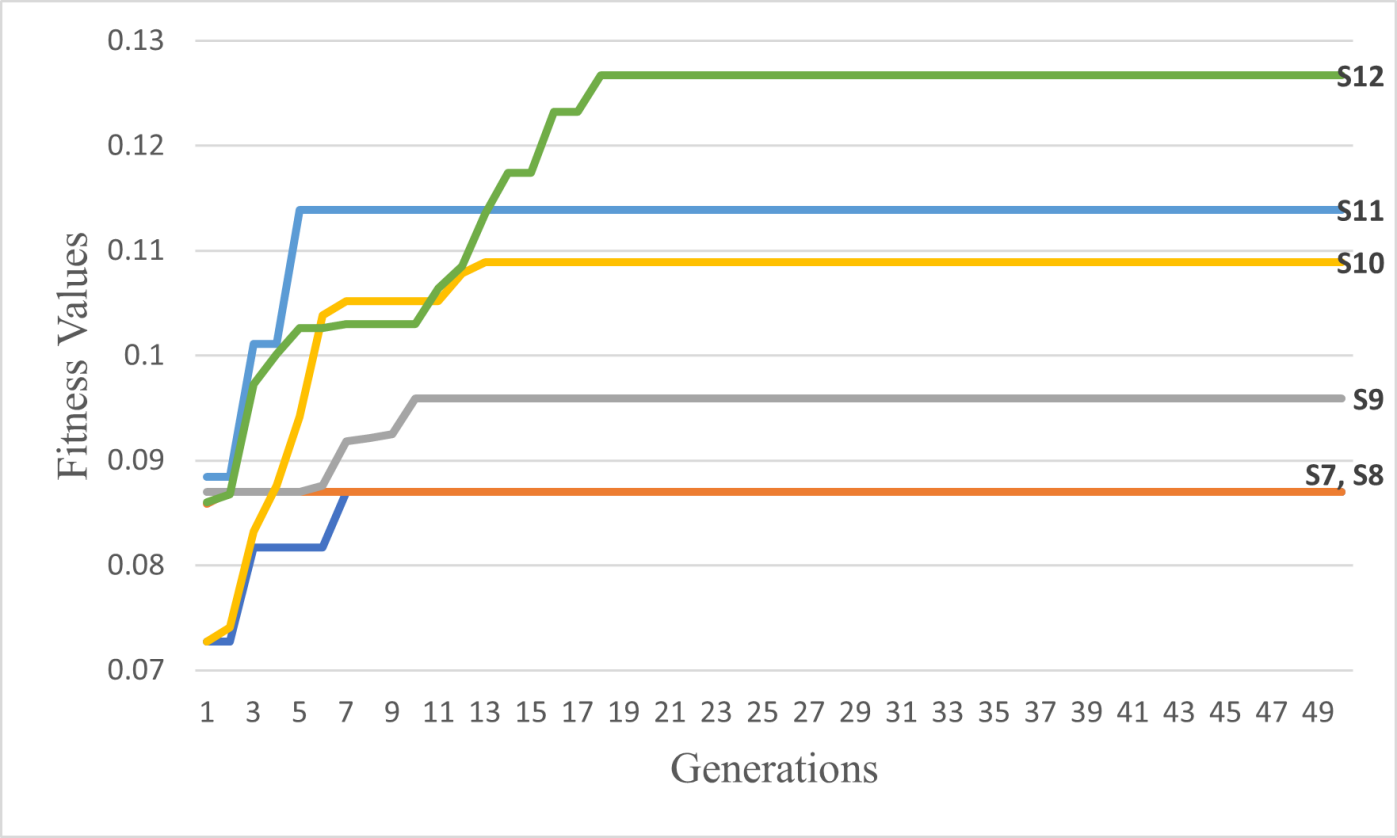
The second six scenarios fitness values.

Then we compared the scenarios relating to their best fitnesses as it is seen in Figs. 17 and 18.

Upon analyzing Fig. 17, the fitness values of S4, S5, and S6 outperform those of S1, S2, and S3, respectively. These results provide strong evidence for the efficiency and accuracy of the suggested COX method in contrast to the CX method. Furthermore, we once again observed that the tournament selection method outperforms the random selection method. Additionally, the modified tournament selection method surpasses the normal tournament selection method. This conclusion is supported by the better fitness values of S2 and S5 compared to S1 and S4, respectively. Moreover, S3 and S6 demonstrate superior fitness values in comparison to S2 and S5, respectively.

Through a comprehensive analysis of Figs. 17 and 18, in addition to reaffirming the conclusions drawn from the first six scenarios, it becomes evident that the suggested fitness function demonstrates remarkable efficiency. This efficiency is particularly evident in the ability to rapidly converge towards the most optimal solution, albeit with some variations influenced by the specific settings of each scenario.

**Figure 19:**
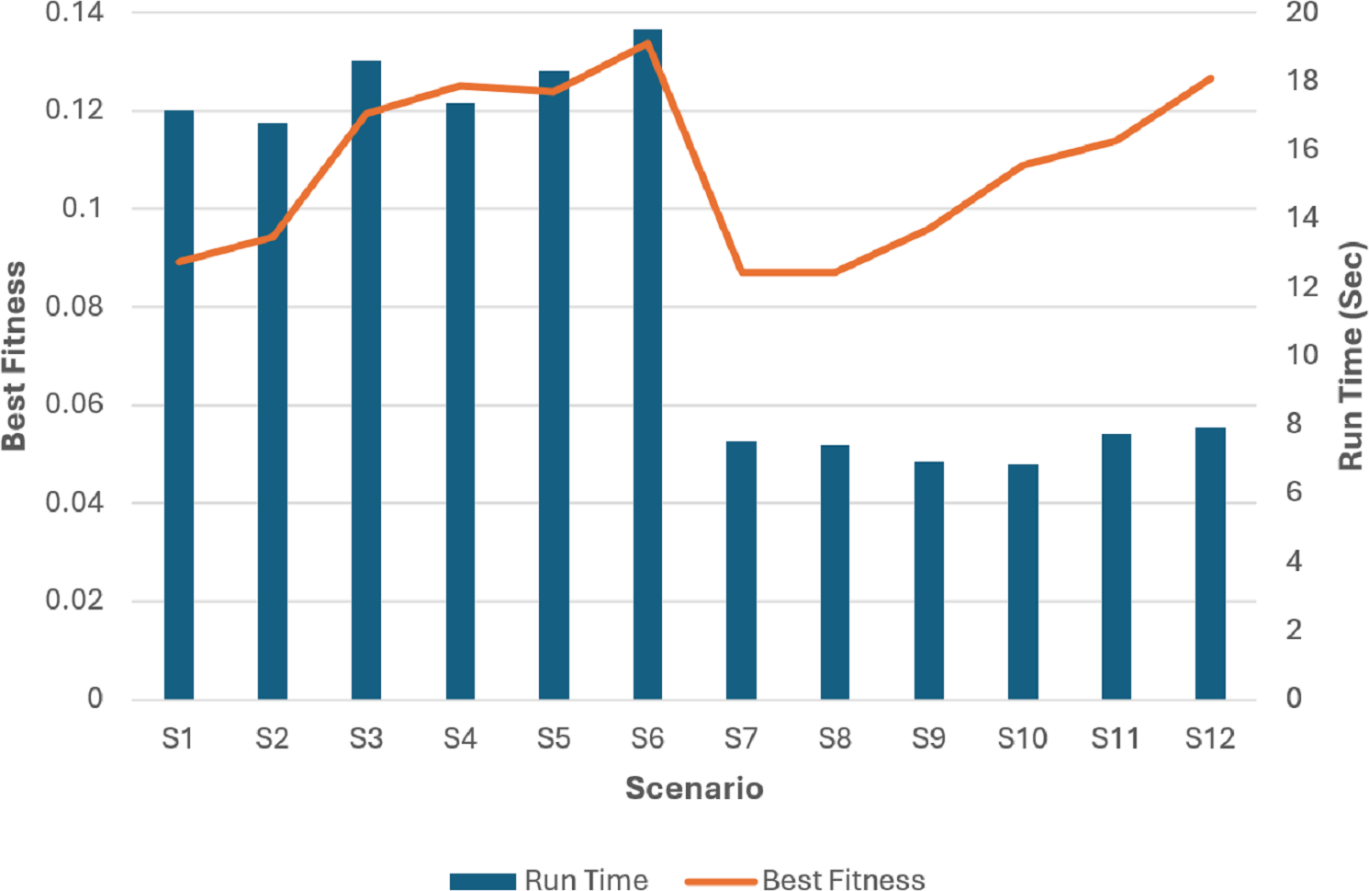
The run time and best fitness for each scenario.

Then we compared the scenarios relating to the running time to get the optimal settings for our problem and got the results in Fig. 19.

The superiority of the modified tournament selection method is evident from the results presented in Fig. 19. The GA scenarios (S3, S6, S9, S12) consistently achieved higher fitness values compared to their corresponding scenarios (S2, S5, S8, S11) respectively.

By analyzing the fitness values depicted in Fig. 19, it can be deduced that the GA scenarios which used our proposed COX method (S4, S5, S6, S10, S11, S12) exhibit superior fitness values in comparison to their corresponding scenarios (S1, S2, S3, S7, S8, S9) which used the classical CX method respectively.

The analysis of Fig. 19 unambiguously establishes the superiority of the S6 and S12 scenarios as the optimal solutions. These scenarios, employing the modified tournament selection method and the proposed COX method, achieved the highest fitness value in the shortest duration, approximately 20 s for S6 and 8 s for S12. In comparison, the 6th scenario, which also employed the same methods but with a larger population size and iteration number, attained slightly better fitness value. However, this achievement required approximately higher computation time.

### Results validity

To statistically validate the superiority of the proposed crossover method (COX) and modified tournament selection method over traditional methods, we employ ANOVA followed by *post-hoc* analysis. This approach tests whether there are statistically significant differences in the performance of GA across different scenarios.

#### Analysis of variances (ANOVA)

Consider the following:

• *S*_1_, *S*_2_, …, *S*_12_ represent the different scenarios described in our experiments.
• *X*_*ij*_ represents the fitness values for the *i*-th scenario (*i* = 1, 2, …, 12) and the *j*-th observation within that scenario (*j* = 1, 2, …, *n*_*i*_).

We use one-way ANOVA to test the null hypothesis *H*_0_: “The means of the fitness values across all scenarios are equal.”

The overall mean ($\overline{X}$) is calculated as follows:

(14) \begin{eqnarray*}\overline{X}= \frac{1}{N} \sum _{i=1}^{k}\sum _{j=1}^{{n}_{i}}{X}_{ij}=0.109116,\end{eqnarray*}

where *N* = 900 is total number of observations in our experiment, *k* = 12 is the number of scenarios, and *n*_*i*_ is the number of observations in the *i*-th scenario.

The Sum of Squares Between Groups (*SSB*) is calculated as follows:

(15) \begin{eqnarray*}SSB=\sum _{i=1}^{k}{n}_{i}{ \left( {\overline{X}}_{i}-\overline{X} \right) }^{2}=0.234,\end{eqnarray*}

where ${\overline{X}}_{i}$ is the mean fitness value for scenario *i*.

The Sum of Squares Within Groups (*SSW*) is calculated as follows:

(16) \begin{eqnarray*}SSW=\sum _{i=1}^{k}\sum _{j=1}^{{n}_{i}}{ \left( {X}_{ij}-{\overline{X}}_{i} \right) }^{2}=0.025.\end{eqnarray*}

Degrees of freedom between groups:

(17) \begin{eqnarray*}df1=k-1=11.\end{eqnarray*}

Degrees of freedom within groups:

(18) \begin{eqnarray*}df2=N-k=900-12=888.\end{eqnarray*}

The Mean Square Between Groups (*MSB*):

(19) \begin{eqnarray*}MSB= \frac{SSB}{df1} .\end{eqnarray*}

The Mean Square Within Groups (*MSW*):

(20) \begin{eqnarray*}MSW= \frac{SSW}{df2} .\end{eqnarray*}

Then the *F*-statistic is:

(21) \begin{eqnarray*}F= \frac{MSB}{SSW} =751.475.\end{eqnarray*}

Our experiment with degrees of freedom between groups (11) and within groups (888) yielded a critical F-value of 1.799 (Soper, 2024). As it is seen in Table 9, the *F*-statistic value (751.475) significantly exceeds this critical value, indicating a statistically significant difference between scenarios (*p* < 0.0001). This rejects the null hypothesis of equal means between scenarios.

**Table 9:** ANOVA test for Istanbul GA scenarios.

	**Sum of squares**	***Df***	**Mean square**	***F*** **-Statistic**	**Sig.**
Between groups	.234	11	.021	751.475	.000
Within groups	.025	888	.000		
Total	.259	899			

#### Post_hoc analysis

**Figure 20:**
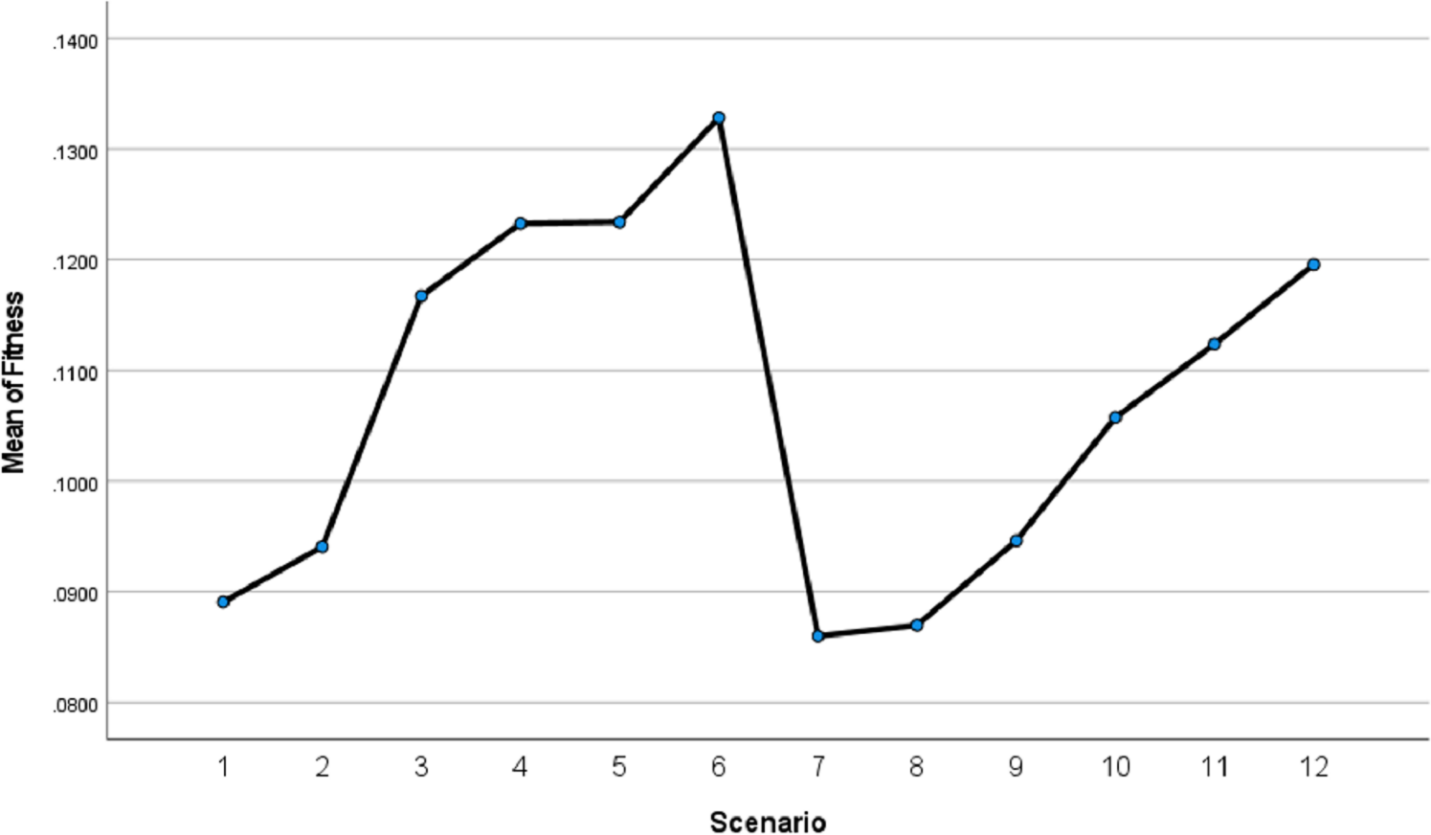
Istanbul GA scenarios fitness values means.

We applied the Tukey Honest Significant Difference (*HSD*) test to determine which specific scenarios differed significantly from each other. The results are presented in Fig. 20.

Figure 20 indicates that scenarios 6, 5, 4, 3, 2, and 1 are ranked in descending order of performance, with scenario 6 achieving the best results compared with others. Similarly, scenarios 12 through 7 follow the same trend. This supports the validity of the results we obtained regarding the superiority of our proposed method.

### Applying GA on the Burma dataset

The quality of our proposed enhanced GA is checked again by applying it on Burma14 dataset as well which consists of 14 places to be visited by a salesman (Reinelt, 1991; Discrete & Combinatorial Optimization Group, 2018). This number of places is close to our dataset number of museums. For applying GA on the Burma dataset, we defined a simple fitness function *F* as the reciprocal of the distance *D* between places:

(22) \begin{eqnarray*}F= \frac{1}{D} .\end{eqnarray*}

Thus, our objective for the Burma dataset problem is to maximize *F* and minimize *D*, similar to the problem for Istanbul. By applying similar GA scenarios, we obtained the results detailed in Figs. S1–S6, and Tables S1 and S2. The key observations include:

• Overall superiority of our proposed approach: By comparing the fitness values of scenarios using our proposed approach (S6 and S12) to those employing the traditional approach (S2 and S8), it is notable that scenario S6 outperforms S2 by 56.60%, and S12 exhibits the same increase in fitness compared to S8. To conclude, that overall enhancement rate in GA using our proposed approach is 56.60% for Burma dataset.
• Highest fitness values for COX scenarios: The GA scenarios S6 and S12, utilizing the proposed COX method, achieved the highest fitness values (0.0368), corresponding to the shortest distance (27.1739 km).
• Modified tournament scenarios superiority: Within each group of scenarios using the same crossover method, the modified tournament selection scenario consistently outperformed the others. For example, the fitness of S3 is higher than that of S1 and S2, and similarly, the fitness of S6 is higher than that of S4 and S5. Additionally, S9 and S12 have the best fitness values within their respective scenario groups (S7, S8, S9) and (S10, S11, S12).
• COX superiority over CX: The COX method scenarios generally achieved higher fitness values compared to the CX method scenarios. For instance, the fitness values for (S4, S5, S6) are superior to those for (S1, S2, S3), and similarly, fitness values for (S10, S11, S12) are superior to those for (S7, S8, S9).

These findings highlight the effectiveness of the COX method and the modified tournament approach in optimizing the GA performance for the Burma dataset. The superiority of our approach is evident, with results comparable to those achieved for the Istanbul dataset.

## Conclusions and Outlook

In conclusion, recent advancements in optimization approaches, particularly genetic algorithm (GA), have revolutionized travel planning. By considering individual constraints such as budget, time, distance, and desired destinations, this GA-based approach can recommend optimal itineraries for tourists. This research has specifically focused on enhancing the efficiency of GA by introducing several modifications and new additions.

Firstly, a new fitness function was proposed to minimize travel time, distance, and cost while ensuring adherence to the specified budget. By incorporating these factors, the algorithm sought to generate optimal solutions tailored to the individual constraints of each tourist.

Furthermore, improvements were made to the tournament selection method and the swap mutation operator to accelerate convergence toward the best fitness values. The tournament selection was modified, and the swap mutation was tuned to maximize fitness within fewer iterations. The experimental results show the superiority of the modified tournament selection.

Additionally, we proposed a new crossover method. COX differs from the already existing order crossover methods, in that it retains the first place by not wrapping, and it doesn’t fill in discrete parts while producing the offspring sequences, as it deals with each part separately and does not make radical changes in the parents’ sequences. Through extensive experimentation, it was demonstrated that this new crossover approach outperformed the traditional cycle crossover CX in all GA execution scenarios, providing more efficient and effective results.

Employing a smaller population size and minimizing the number of generations in our enhanced GA approach accelerated the algorithm run time by approximately 2.5 times compared to the traditional GA scenarios.

### Limitations and outlook

This study acknowledges limitations associated with considering only travel factors (cost, time, and distance) for itinerary recommendation. These factors may not exhibit linear relationships with user preferences. For instance, budget-conscious backpackers might prioritize affordability even with longer travel times and less comfortable accommodations, while luxury travelers might value shorter travel times and high-end experiences. Another limitation lies in the potential for user preferences to evolve throughout the itinerary. For example, a tourist initially planning outdoor activities might shift towards museums or indoor attractions due to unexpected bad weather. A static GA might struggle to capture these nuances. To address these limitations, we propose investigating the integration of Multivariate Adaptive Regression Splines (MARS) with the GA framework for itinerary recommendation. MARS can potentially capture the non-linear relationships between travel factors and user preferences, while the GA’s optimization capabilities can be leveraged to generate efficient itineraries that adapt to dynamic user preferences.

#### MARS for itinerary recommendation

MARS is a non-parametric regression technique developed by Friedman (1991) for modeling complex relationships in data. MARS achieves this by automatically identifying nonlinearities and interactions between variables. MARS-based approach is used in various fields, including addressing data uncertainty and optimizing natural gas consumption models by Özmen, Zinchenko & Weber (2023), approximating data in stochastic systems for studying the mutual relationship between financial processes and investors’ sentiment by Kalaycı, Özmen & Weber (2020), and using magnetic resonance images for detecting diseases (Çevik et al., 2017).

Utilizing MARS for itinerary recommendations has many advantages like:

1. Complex relationship modeling: MARS is adept at identifying non-linearities and interactions between variables, making it ideal for analyzing user data (past trips, preferences, demographics) and travel information (destination details, activity costs, weather patterns). This enables suggestions that go beyond basic user input.

2. Data type versatility: MARS can handle various data types, which is crucial for capturing the subjective and complex nature of travel preferences influenced by factors such as budget constraints, travel style, and weather impact on activities. This allows MARS to generate more nuanced itineraries.

3. Unexpected connections: MARS can uncover unexpected connections within the data. For instance, it might reveal that users who enjoy hiking in a specific region are also interested in local historical sites, even if these interests aren’t explicitly linked. This helps to suggest relevant activities that users might not have considered.

#### Mathematical insights

MARS constructs models that can capture complex, non-linear relationships within the data by fitting piecewise linear splines. The MARS model, adapted for itinerary recommendation, is formulated as follows:

(23) \begin{eqnarray*}\hat {S} \left( I \right) ={\beta }_{0}+\sum _{m=1}^{M}{\beta }_{m}{h}_{m}({X}_{I}),\end{eqnarray*}

where:

• $\hat {S} \left( I \right) $ represents the predicted user satisfaction for itinerary *I*.
• *β*_0_ is the intercept term.
• *β*_*m*_ are the coefficients associated with each basis function.
• *h*_*m*_(*X*_*I*_) are the basis functions applied to the vector of travel factors *X*_*I*_ relevant to itinerary.
• *M* is the total number of basis functions used in the model.

The basis functions *h*_*m*_(*X*_*I*_) in the MARS model are of two types: Hinge functions and product of hinge functions.

1. Hinge functions: Hinge functions help to identify thresholds, such as user preferences for shorter travel distances for weekends. The mathematical representation is:

(24) \begin{eqnarray*}h \left( x,t \right) =(x-t)_{+}=\max \nolimits \left( 0,x-t \right) ;\end{eqnarray*}

and

(25) \begin{eqnarray*}h \left( x,t \right) =(t-x)_{+}=\max \nolimits \left( 0,t-x \right) ,\end{eqnarray*}

where *x* is the input value (*e.g.*, distance), and *t* is a knot or threshold value (*e.g.*, a distance value specific to weekend trips).

2. Product of hinge functions: To model interactions between travel vectors, the MARS model can also use the product of hinge functions. This product allows the model to capture the combined effect of multiple factors on user satisfaction. The Product of Hinge Functions is defined as:

(26) \begin{eqnarray*}h \left( {x}_{1},{t}_{1},{x}_{2},{t}_{2} \right) =\max \nolimits \left( 0,{x}_{1}-{t}_{1} \right) \times \max \nolimits \left( 0,{x}_{2}-{t}_{2} \right) ,\end{eqnarray*}

where:

• *x*_1_ and *x*_2_ are different travel factors (*e.g.*, cost and time).
• *t*_1_ and *t*_2_ are the corresponding kont points.

For instance, if *x*_1_represents cost and *x*_2_ represents time, $h \left( {x}_{1},{t}_{1},{x}_{2},{t}_{2} \right) $ could model how user satisfaction decreases when both the cost and time exceed their respective preferred thresholds *t*_1_ and *t*_2_.

Combining the hinge functions and their products, the basis functions *h*_*m*_(*X*_*I*_) in the MARS model can be expressed as:

(27) \begin{eqnarray*}{h}_{m} \left( {X}_{I} \right) =\prod _{k=1}^{{K}_{m}}\max \nolimits \left( 0,{x}_{{i}_{k}}-{t}_{{i}_{k}} \right) ,\end{eqnarray*}

where:

• *K*_*m*_ is the number of hinge functions involved in the product for the *m*-th basis function.
• *x*_*i*_*k*__ is the *k*-th travel factor in the itinerary *I*.
• *t*_*i*_*k*__ is the corresponding knot point.

This formulation allows the MARS model to capture both the individual effects of travel factors and their interactions on user satisfaction. It ensures that the model can accurately predict user preferences for itineraries based on a combination of factors like cost, time, and distance.

MARS two-step process: To identify the most relevant basis functions ${h}_{m} \left( {X}_{I} \right) $, MARS employs a two-step process: forward selection and backward elimination.

1. Forward selection: MARS incrementally adds basis functions that most reduce the residual error and improve the model’s accuracy. The performance of the model at each step is evaluated using the residual sum of squares (*RSS*):

(28) \begin{eqnarray*}RSS=\sum _{i=1}^{n}({y}_{i}-{\hat {y}}_{i})^{2},\end{eqnarray*}

where *y*_*i*_ is the actual satisfaction score for itinerary *I*_*i*_, ${\hat {y}}_{i}$ is the predicted satisfaction score, and *n* is the number of itineraries.

The model at this stage is defined as:

(29) \begin{eqnarray*}{\hat {S}}_{forward} \left( I \right) ={\beta }_{0}+\sum _{m=1}^{{M}_{f}}{\beta }_{m}{h}_{m}({X}_{I}),\end{eqnarray*}

where *M*_*f*_ is the number of basis functions after forward selection.

2. Backward elimination: MARS then removes less significant basis functions to streamline the model while maintaining its predictive power. This step prevents overfitting. The effectiveness of the pruning process is assessed using the generalized cross-validation (*GCV*) criterion:

(30) \begin{eqnarray*}GCV= \frac{RSS}{(n-pM)^{2}} ,\end{eqnarray*}

where *p* is a penalty term (related to the complexity of the model), *M* is the number of basis functions, and *n* is the number of data points.

The final model is given by:

(31) \begin{eqnarray*}\hat {S} \left( I \right) ={\beta }_{0}+\sum _{m=1}^{{M}_{b}}{\beta }_{m}{h}_{m}({X}_{I}),\end{eqnarray*}

where *M*_*b*_ (with *M*_*b*_ ≤ *M*_*f*_) is the number of basis functions retained after backward elimination.

The *RSS* measures the difference between the actual and predicted satisfaction scores, helping the model identify the best-fitting basis functions during forward selection. The *GCV*, on the other hand, helps prevent overfitting during backward elimination by penalizing models that are too complex. These metrics ensure that only the most relevant factors, which significantly affect user satisfaction, are included in the final model.

#### Integrating MARS with GA

Once MARS has revealed these hidden patterns and potentially generated new features (*e.g.*, ”adjusted_distance” based on weather), GA can optimize these features within the itinerary recommendation process.

1. Itinerary representation: Each potential itinerary *I* is represented as an individual in the GA population. This includes chosen flights, hotels, activities, and their associated costs *C* (*I*), travel times *T* (*I*), distances *D* (*I*), and satisfaction scores $\hat {S} \left( I \right) $ predicted by MARS.

2. Fitness function: A fitness function *F* (*I*) is a weighted combination of factors like:

(32) \begin{eqnarray*}F \left( I \right) ={w}_{c}C \left( I \right) +{w}_{t}T \left( I \right) +{w}_{d}D \left( I \right) +{w}_{s}\hat {S} \left( I \right) ,\end{eqnarray*}

where *w*_*c*_, *w*_*t*_, *w*_*d*_, and*w*_*s*_ are weights for cost, travel time, distance, and satisfaction score, respectively.

3. GA iteration: The GA iteratively improves the population of itineraries, selecting those that offer the best combination of high satisfaction and low costs, travel time, and distance.

This integration allows the system to make recommendations that accurately reflect detailed user preferences and uncover hidden patterns in the data, thereby significantly enhancing the travel planning experience.

## Supplemental Information

10.7717/peerj-cs.2340/supp-1 Supplemental Information 1 Burma GA results

10.7717/peerj-cs.2340/supp-2 Supplemental Information 2 Burma best itineraries graphs of scenarios S1 through S6 (A) GA Scenarios with CX crossover; (B) GA Scenarios with COX crossover.

10.7717/peerj-cs.2340/supp-3 Supplemental Information 3 Burma best itineraries graphs of scenarios S7 through S12 (A) GA Scenarios with CX crossover; (B) GA Scenarios with COX crossover.

10.7717/peerj-cs.2340/supp-4 Supplemental Information 4 Burma itineraries fitness values of GA first 6 scenarios

10.7717/peerj-cs.2340/supp-5 Supplemental Information 5 Burma itineraries fitness values of GA second 6 scenarios

10.7717/peerj-cs.2340/supp-6 Supplemental Information 6 GA run time and best fitness for each scenario of Burma

10.7717/peerj-cs.2340/supp-7 Supplemental Information 7 ANOVA Test on Burma GA Scenarios

10.7717/peerj-cs.2340/supp-8 Supplemental Information 8 Burma GA Scenarios Fitness Values Means

10.7717/peerj-cs.2340/supp-9 Supplemental Information 9 Code and Data (A) “Turism_Istanbul” folder includes the Matlab code files in addition to Istanbul 12 Museums coordinates dataset as a mat file. The results figures are included as well. “Turism_Burma” folder includes Burma 14 places Dataset as well in addition to the matlab code files and results figures of applying GA on Burma. (B) Changing Generations number and Population size can be done in “Main_prog.m” file. (C) The selection method can be chosen in the “GA.m” file by activating one method and dis-activating others. (D) The crossover method can be chosen in “GA.m” file in the “% Perform Crossover” section by recalling either COX or CX function. DOI: 10.5281/zenodo.12736186

## Additional Information and Declarations

### Data Availability

The following information was supplied regarding data availability: The Istanbul and Burma datasets are available in the Supplemental Files. The source code and raw data are available at GitHub and Zenodo: - https://github.com/MuhammedSehab/GA-for-Itinerary-Planning
- MuhammedSehab. (2024). MuhammedSehab/GA-for-Itinerary-Planning: Initial Release (0.1.0). Zenodo. https://doi.org/10.5281/zenodo.12736186.
