# Supplementary material for: An enhanced genetic algorithm solution for itinerary recommendation considering various constraints
Source: PeerJ Comput Sci. 2024 Oct 2;10:e2340. doi: 10.7717/peerj-cs.2340 (PMC11623113; doi:10.7717/peerj-cs.2340)
Supplement: Supplemental Information 1 [file peerj-cs-10-2340-s001.docx]

| **Scenario** | **Best Fitness** | **First Generation Number Which Reached Best Fitness** | **GA Run Time (Sec)** |
| --- | --- | --- | --- |
| S1 | 0.0235 | 1^st^ | 4.5185 |
| S2 | 0.0235 | 1^st^ | 4.8291 |
| S3 | 0.0311 | 37^th^ | 9.5948 |
| S4 | 0.0333 | 17^th^ | 4.5108 |
| S5 | 0.0354 | 4^th^ | 5.1255 |
| S6 | 0.0368 | 11^th^ | 5.9336 |
| S7 | 0.0235 | 1^st^ | 1.3949 |
| S8 | 0.0235 | 1^st^ | 1.4637 |
| S9 | 0.0309 | 45^th^ | 2.8506 |
| S10 | 0.0320 | 32^nd^ | 1.5062 |
| S11 | 0.0322 | 5^th^ | 2.7522 |
| S12 | 0.0368 | 44^th^ | 2.9310 |
