# Supplementary figures and images for: An enhanced genetic algorithm solution for itinerary recommendation considering various constraints
Source: PeerJ Comput Sci. 2024 Oct 2;10:e2340. doi: 10.7717/peerj-cs.2340 (PMC11623113; doi:10.7717/peerj-cs.2340)

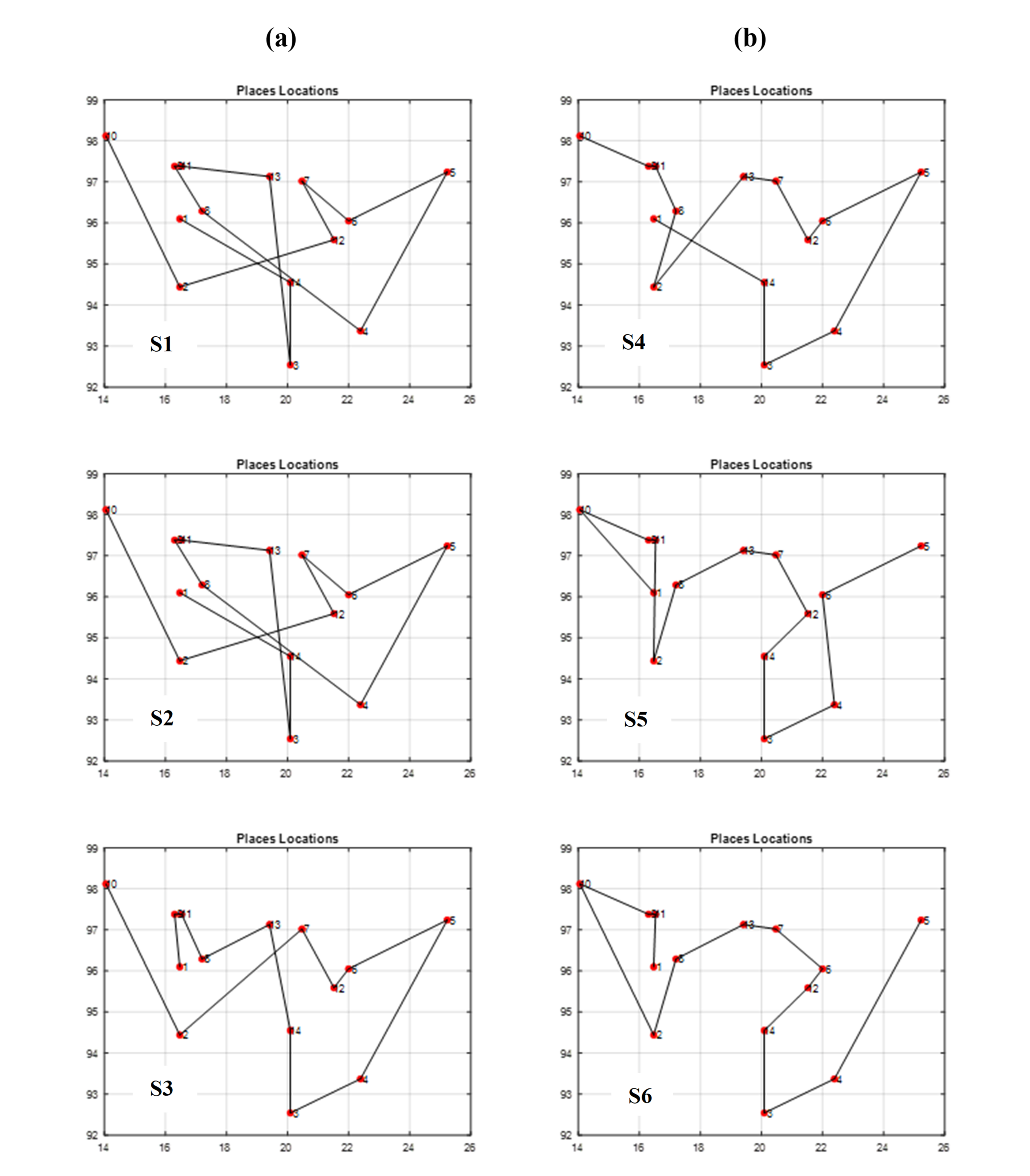

Supplement: Supplemental Information 2 — (A) GA Scenarios with CX crossover; (B) GA Scenarios with COX crossover. [file peerj-cs-10-2340-s002.png]

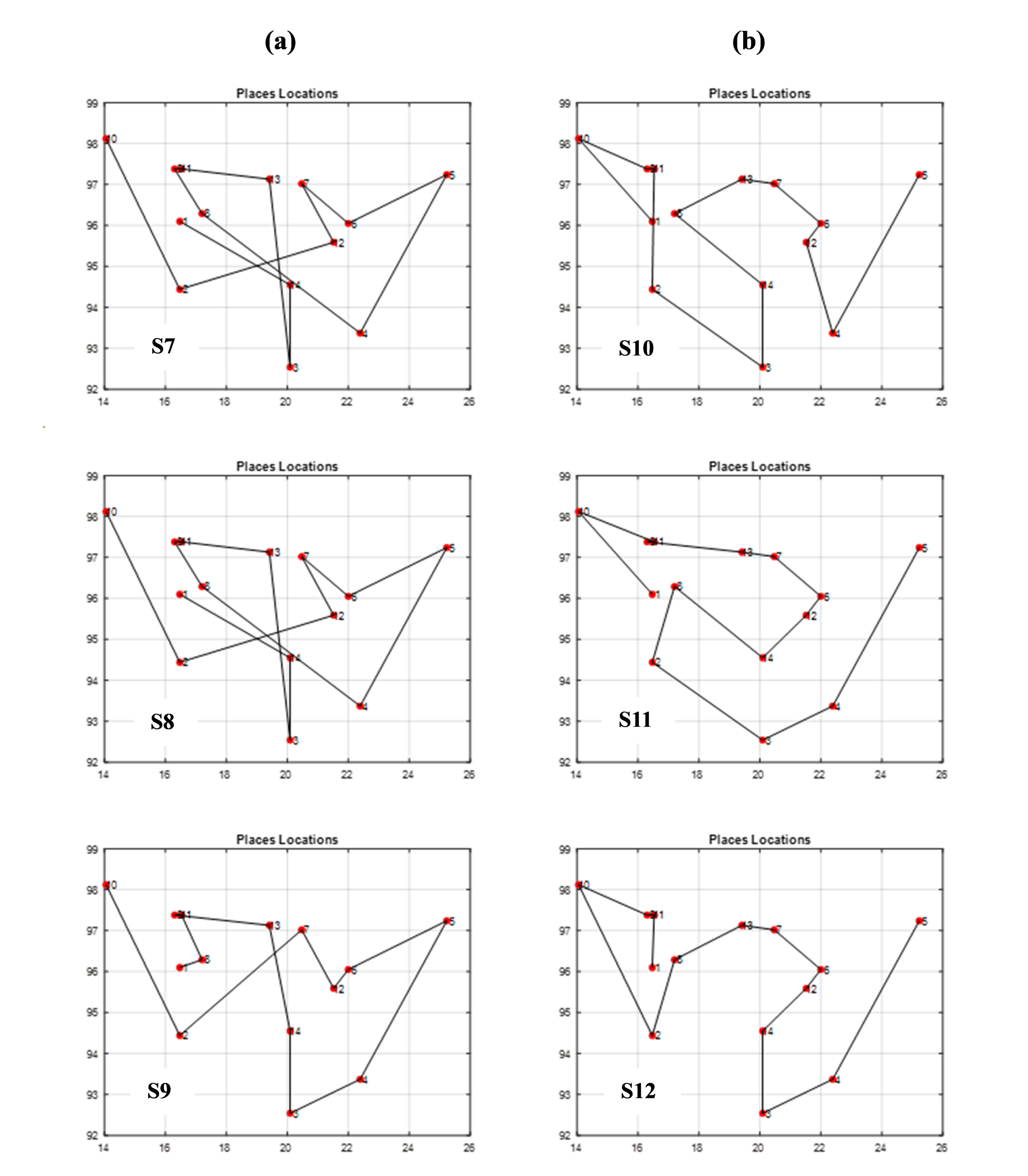

Supplement: Supplemental Information 3 — (A) GA Scenarios with CX crossover; (B) GA Scenarios with COX crossover. [file peerj-cs-10-2340-s003.png]

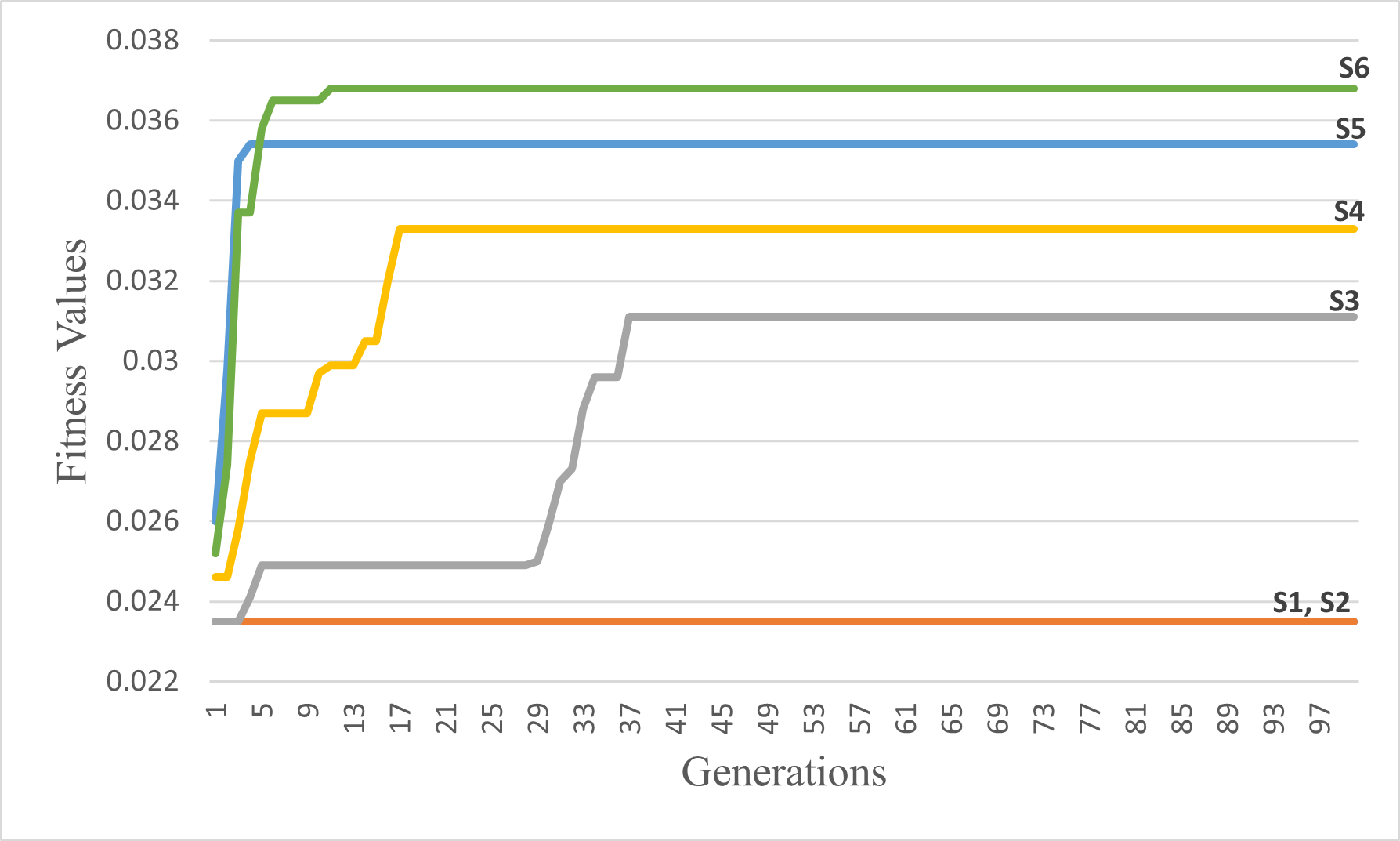

Supplement: Supplemental Information 4 [file peerj-cs-10-2340-s004.png]

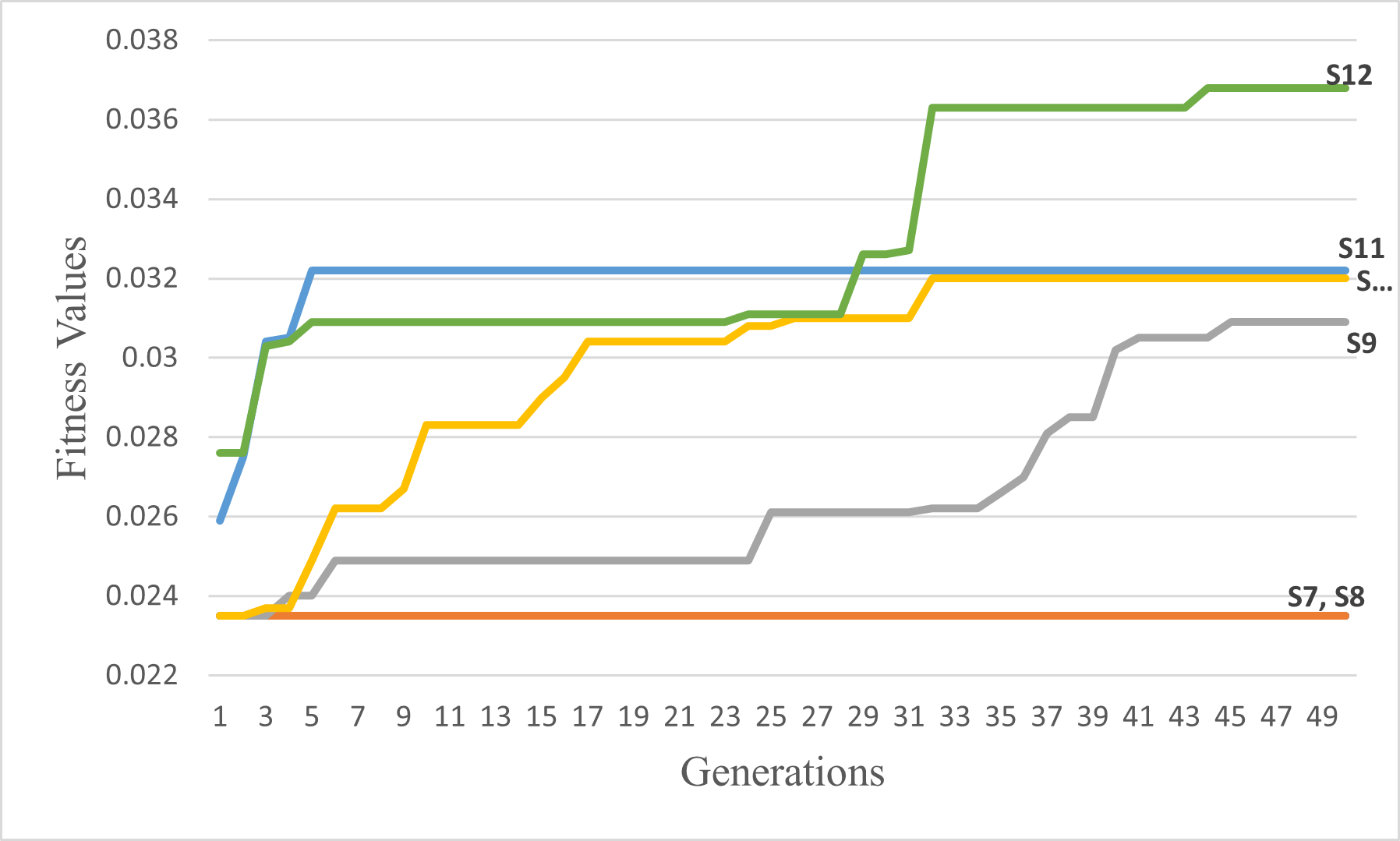

Supplement: Supplemental Information 5 [file peerj-cs-10-2340-s005.png]

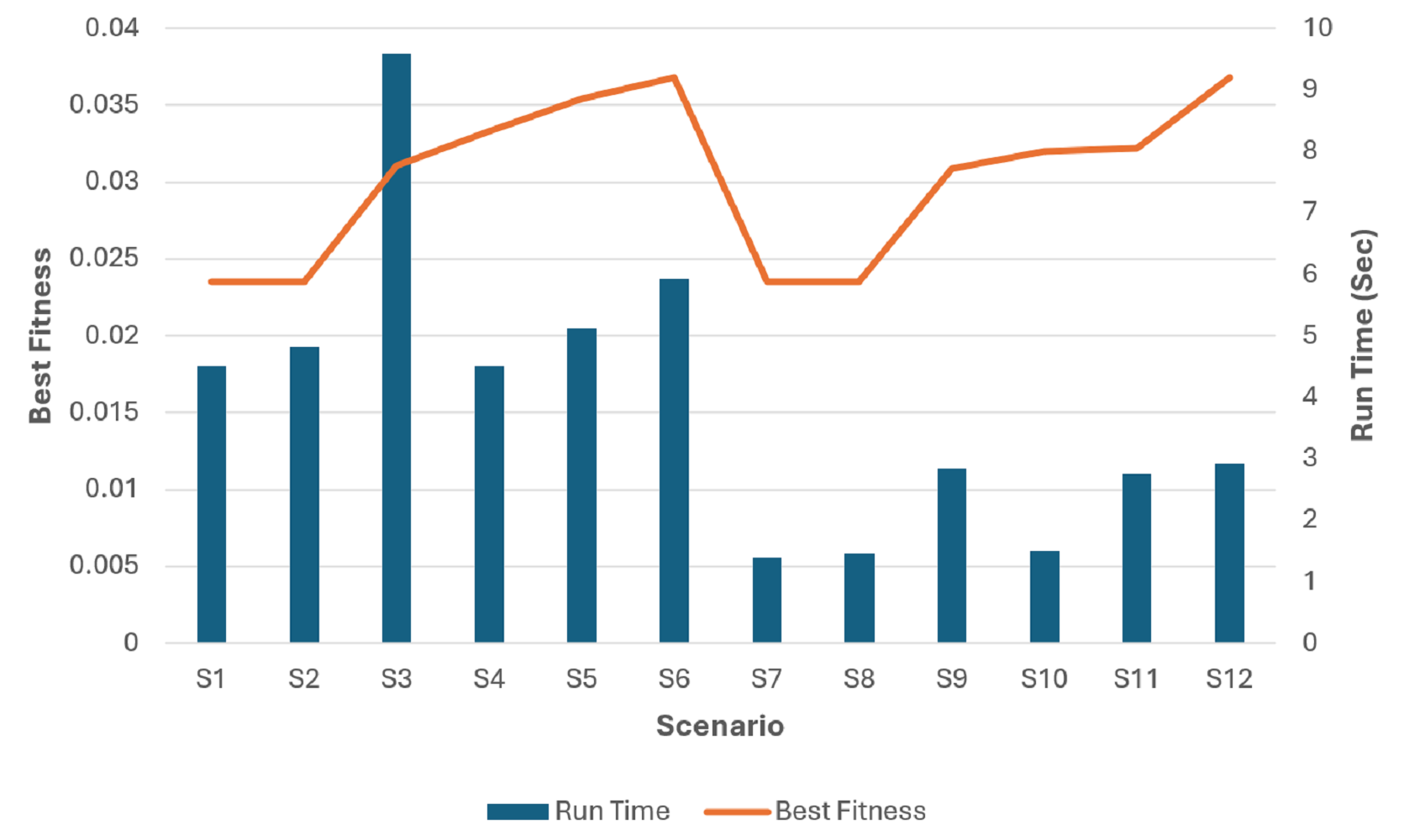

Supplement: Supplemental Information 6 [file peerj-cs-10-2340-s006.png]

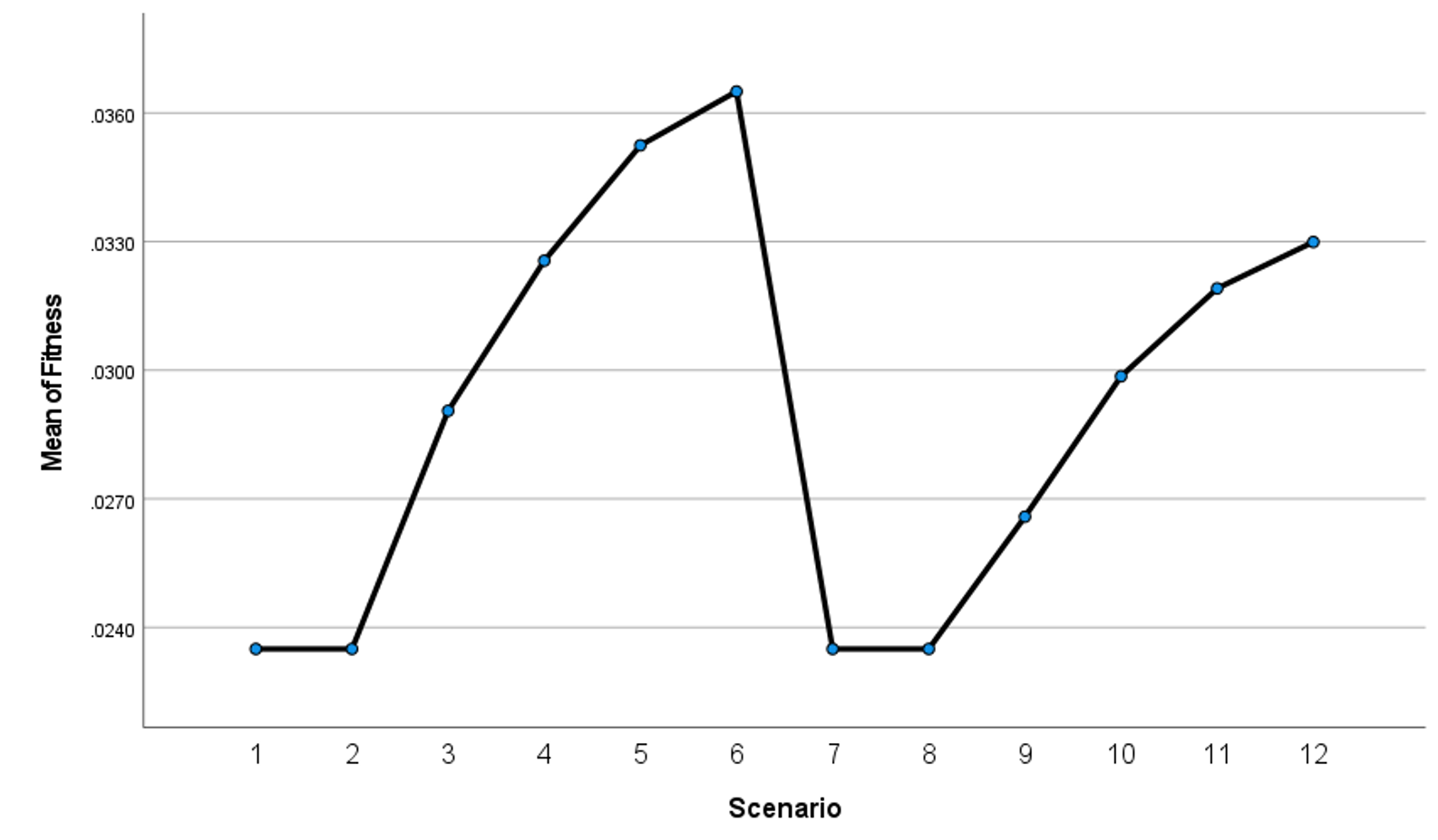

Supplement: Supplemental Information 8 [file peerj-cs-10-2340-s008.png]
