# Supplementary material for: An enhanced genetic algorithm solution for itinerary recommendation considering various constraints
Source: PeerJ Comput Sci. 2024 Oct 2;10:e2340. doi: 10.7717/peerj-cs.2340 (PMC11623113; doi:10.7717/peerj-cs.2340)
Supplement: Supplemental Information 7 [file peerj-cs-10-2340-s007.docx]

|  | **Sum of Squares** | ***Df*** | **Mean Square** | ***F*-Statistic** | **Sig.** |
| --- | --- | --- | --- | --- | --- |
| Between Groups | .021 | 11 | .002 | 648.305 | .000 |
| Within Groups | .003 | 888 | .000 |  |  |
| Total | .024 | 899 |  |  |  |
